# The Manifold Cellular Functions of von Willebrand Factor

**DOI:** 10.3390/cells10092351

**Published:** 2021-09-08

**Authors:** Angelika Mojzisch, Maria A. Brehm

**Affiliations:** 1 Dermatology and Venerology, University Medical Center Hamburg-Eppendorf, 20246 Hamburg, Germany; a.mojzisch@uke.de; 2 School of Life Sciences, University of Siegen, 57076 Siegen, Germany

**Keywords:** von Willebrand factor, angiogenesis, integrin αvβ3, GPIbα, apoptosis, metastasis

## Abstract

The plasma glycoprotein von Willebrand factor (VWF) is exclusively synthesized in endothelial cells (ECs) and megakaryocytes, the precursor cells of platelets. Its primary function lies in hemostasis. However, VWF is much more than just a “fishing hook” for platelets and a transporter for coagulation factor VIII. VWF is a true multitasker when it comes to its many roles in cellular processes. In ECs, VWF coordinates the formation of Weibel–Palade bodies and guides several cargo proteins to these storage organelles, which control the release of hemostatic, inflammatory and angiogenic factors. Leukocytes employ VWF to assist their rolling on, adhesion to and passage through the endothelium. Vascular smooth muscle cell proliferation is supported by VWF, and it regulates angiogenesis. The life cycle of platelets is accompanied by VWF from their budding from megakaryocytes to adhesion, activation and aggregation until the end in apoptosis. Some tumor cells acquire the ability to produce VWF to promote metastasis and hide in a shell of VWF and platelets, and even the maturation of osteoclasts is regulated by VWF. This review summarizes the current knowledge on VWF’s versatile cellular functions and the resulting pathophysiological consequences of their dysregulation.

## 1. Introduction

**Figure 1:**
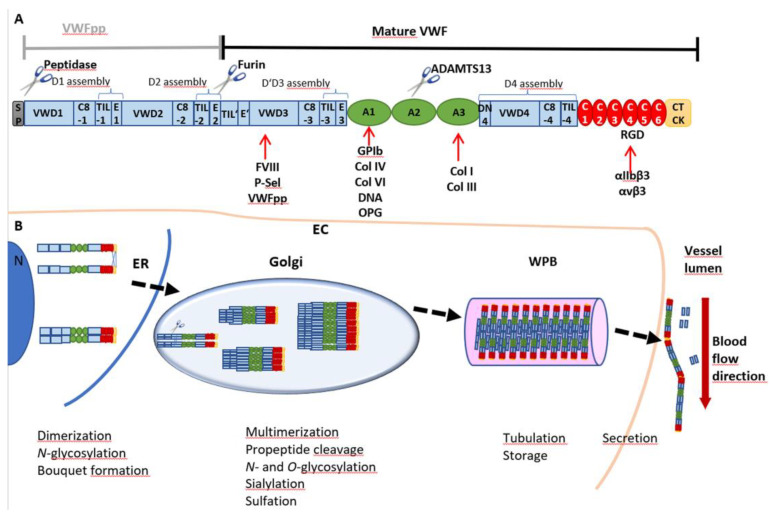
VWF domain structure and multimer biosynthesis. (**A**) VWF is synthesized as a pre-pro-monomer with a signal peptide (SP) and a pro-peptide (VWFpp) containing the D1 and D2 assemblies, each consisting of a VWD, C8, TIL and E domain. The VWFpp is cleaved off by furin, leaving the mature VWF, including the D’D3 assembly and domains A1, A2 and A3, followed by the D4 assembly, C1-C6 and the CTCK domains. Cleavage and thus degradation of VWF can occur between Tyr1605 and Met1606 of the A2 domain by a disintegrin and metalloproteinase with a thrombospondin type 1 motif, member 13 (ADAMTS13). Binding partners are indicated below the respective domains: coagulation factor VIII (FVIII), P-selectin (P-sel) and the VWFpp interact with D’D3; the A1 domain is the binding site for glycoprotein Ibα (GPIbα), collagens (Col) IV and VI, DNA and osteoprotegerin (OPG); collagens I and III bind to A3; and the RGD motif in C4 is the interaction site for integrins αIIbβ3 (= receptor GPIIb/IIIa) and αvβ3. (**B**) The SP guides translation into the endoplasmic reticulum (ER), where monomers are dimerized by the formation of three disulfide bonds between the CTCK domains. Further *N*-glycosylation is initiated, and dimers arrange into a “bouquet-like” formation with a closed stem region. In the Golgi apparatus, dimers are multimerized by disulfide bonds between D’D3 domains, and VWF is heavily glycosylated, sialylated and sulfated. The VWFpp is cleaved but stays non-covalently attached and supports multimerization and, later on, tubule formation, packaging and storage in Weibel–Palade bodies (WPB). After secretion into the vessel lumen, the VWFpp is released.

Von Willebrand factor (VWF) is a multimeric glycoprotein found in the peripheral blood stream. It serves as an essential driver of primary hemostasis as it induces platelet adhesion and plug formation [1]. Expression of VWF takes place exclusively in endothelial cells (ECs) (Figure 1) [2] and megakaryocytes, the progenitor cells of platelets [3]. Before secretion into the circulation, VWF undergoes a highly complex cascade of posttranslational modifications until high-molecular weight multimers (HMWM) are fully formed (Figure 1B). The biosynthesis of VWF starts with a 2813-amino acid (aa) pre-pro-monomer [4], which consists of a 22 aa signal peptide, a 741 aa pro-peptide (VWFpp) and the mature VWF with 2050 aa [5]. The signal peptide directs VWF to the membrane of the endoplasmic reticulum (ER) during protein translation. The remaining pro-VWF monomer is a multidomain protein composed of a repetitive sequence of domains: D1-D2-D’D3-A1-A2-A3-D4-C1-C2-C3-C4-C5-C6-CK (Figure 1A) [6]. The D’D3 domain harbors binding sites for coagulation factor VIII (FVIII), P-selectin (P-sel) and the VWFpp. Platelet glycoprotein (GP)Ibα, DNA, osteoprotegerin (OPG) and collagens IV and VI bind to A1, whereas binding sites for collagens I and III are located in A3. The RGD motif in the C4 domain is the interaction site for integrins αIIbβ3 (= receptor GPIIb/IIIa) and αvβ3.

VWF monomers are dimerized in the ER through disulfide bonds between the CK domains in a “tail-to-tail” manner. Protein disulfide isomerase (PDI) isoform A1 catalyzes the formation of two intermolecular bonds between Cys2771 and Cys2773 which facilitates a third bond between residues Cys2811 of the two monomers [7]. The dimers arrange into “bouquet-like” structures, in which the C-terminal domains of the two VWF monomers are closely associated with each other into a stem region [8]. Dimers are translocated to the Golgi apparatus where multimerization is catalyzed by VWF’s pro-peptide itself as its oxidoreductase activity is switched on by the lower pH in the Golgi [9]. Thereby, dimers are connected by disulfide linkage between the D’D3 domains in a “head-to-head” manner [10]. Meanwhile, the pro-peptide consisting of the D1-D2 assemblies is cleaved off by furin but stays non-covalently attached to the mature VWF [11]. During maturation, VWF is further decorated by extensive *N*- and *O*-glycosylation, sialylation [12,13,14,15] and sulfation [16] (Figure 1B). The newly synthesized VWF is either secreted basolaterally by ECs as low-molecular weight (LMW) VWF or packaged into specific storage organelles, called Weibel–Palade bodies (WPBs) [17]. From WPBs, secretion of ultra-large VWF (UL-VWF) predominantly occurs apically via basal as well as regulated secretory pathways. Basally secreted VWF is released continuously from WPBs in the absence of stimuli to maintain a constant plasma antigen level of VWF [17]. Stimulated secretion, for example, by histamine [18], thrombin [19], epinephrine [20] or vasopressin [21] facilitates VWF to fulfill its role in platelet recruitment to the vessel wall [17]. In contrast to storage in ECs, VWF in megakaryocytes and platelets is stored in α-granules [22]. 

In the circulation, VWF exhibits two main functions. First, VWF is involved in secondary hemostasis as it binds and serves as a carrier for coagulation factor VIII (FVIII), protecting it from degradation. Second, it serves as a sensor for vessel damage and recruits platelets to the side of injury, thereby initiating primary hemostasis [23]. Damage of the endothelium is linked to exposure of extracellular matrix proteins, such as collagen. VWF is able to recognize different types of collagens as it possesses binding sites for collagens I and III in the A3 domain [24] and for collagens IV and VI in its A1 domain [25,26] (Figure 1A). Upon binding, VWF becomes anchored to the vessel wall, and the resulting higher hydrodynamic forces acting on the mechanosensitive VWF lead to stretching of the molecule and opening of its A1 domain. This allows platelets to bind via glycoprotein GPIbα [27]. This interaction will initiate activation of platelets and lead to the formation of an interconnected network stabilized by the crosslinking of the now activated platelet receptor GPIIb/IIIa (=αIIbβ3), VWF, fibrin(ogen) and other clot proteins to prevent prolonged blood loss [28]. Thus, the activity of VWF is strongly dependent on its extent of multimerization as larger VWF molecules display a higher GPIbα binding capacity. This is particularly evident by the fact that dysfunction of VWF and loss of HMWM lead to the most common hereditary bleeding disorder, called von Willebrand disease (VWD). Three main VWD subtypes are distinguished by quantitative defects in VWF (type 1), qualitative defects (type 2) and almost complete absence of plasmatic VWF (type 3) [29]. In contrast, excessive activity of VWF and enhanced aggregation may also promote diseases by increasing the risk of thromboembolic events [30,31]. To counteract this pathophysiological state, regulation of VWF multimer size is necessary. Similar to the process of its activation, VWF cleavage is also regulated by mechanical forces. The VWF-specific protease ADAMTS13 (a disintegrin and metalloproteinase with a thrombospondin type 1 motif, member 13) can cleave VWF between aa residues Tyr1605 and Met1606, but only when its A2 domain is present in a stretched and opened conformation [32]. Oxidation and sulfation of Met1606 have further been described to modulate this process. In vitro oxidation of this amino acid residue to methionine sulfoxide by peroxynitrite [33] and by HOCl was shown to inhibit ADAMTS13 cleavage [34]. This process is accelerated by shear stress-induced unfolding of VWF [35]. In vivo, chronic kidney disease is accompanied by high oxidative stress which can lead to Met1606 oxidation and thus accumulation of prothrombotic ultra-large VWF, increasing the risk for thrombotic and septic complications in these patients [36].

Besides the well-known functions of VWF in hemostasis, more and more data emerge indicating functions in cell signaling, proliferation and apoptosis. In this review, we summarize the currently known functions of VWF in different cell types, thereunder ECs, megakaryocytes, platelets, vascular smooth muscle cells and osteoclasts as well as cancer and immune cells. 

## 2. Cellular Functions of VWF

### 2.1. VWF and Endothelial Cells

#### 2.1.1. Regulated VWF Gene Expression

The expression of VWF is restricted to ECs and megakaryocytes. Thus, a tight regulation of VWF gene expression is needed to ensure expression only in these cell types. VWF is encoded in 52 exons on chromosome 12. The transcription start site (TSS) is located in exon 1, which has a length of 250 bp. Translation of the VWF protein starts at the methionine start codon found in exon 2. Investigation of the 5′ untranslated region (UTR) of the VWF gene in ECs revealed several cis-acting elements and binding sites for transcription factors. The cell type-specific promotor region identified by Jahroudi and Lynch spans the sequence from −487 to +247 relative to the TSS and contains several regulatory elements. The authors annotated the region from −90 to +22 to be the minimal core promotor which is sufficient to activate gene expression in a non-cell-type-specific manner. Further, a negative regulatory element is present upstream at −487 to −313 which diminishes the activation of gene transcription in all cell types. A counteractive positive regulatory element was identified in position +155 to +247 located in exon 1 which reactivates expression in the respective cells [37]. Further analyses identified specific binding sites for transcription factors in the promotor region, such as the positive regulators GATA binding proteins 2, 3 and 6, transcription factors of the Ets family and Histone H1-like protein [38,39,40], as well as the negative regulators nuclear factor I (NFI) [41] and octamer-binding protein 1 (Oct-1) [42]. An additional transcription factor, namely, nuclear factor Y (NFY), was identified to act as an activator or repressor depending on its DNA binding site [43]. Interestingly, further regulatory elements were found upstream of the mentioned promotor, including a GT repeat element, which is activated at physiologically relevant levels of shear stress, thereby enhancing the VWF promotor activity by flow-induced responsiveness [44].

Besides the influence of promotors and repressors on the transcription of VWF, epigenetic modifications were also found to impact VWF expression. Shirodkar et al. demonstrated that the VWF promotor is nearly completely unmethylated in human umbilical vein endothelial cells (HUVECs), human microvascular endothelial cells (HMVECs) and blood outgrowth endothelial cells (BOECs), in contrast to the high levels of methylation in non-endothelial cells, such as human aortic vascular smooth muscle cells (HuAoVSMCs), keratinocytes or hepatocytes [45]. Accordingly, other studies also correlated decreased VWF expression with hypermethylation of the VWF promoter [46,47]. Besides demethylation of DNA, histone acetylation was also found to mediate promotor activity. The transcription inhibitor NFY interacts with histone deacetylases (HDACs) and recruits them to the VWF promotor. Deacetylation of the histone H4 takes place and inhibits promotor activation of VWF [48]. Vice versa, Nakhaei-Nejad et al. investigated HUVECs and EC-differentiated induced pluripotent stem cells and found that histone acetylase P300/CBP-associated factor (PCAF) is guided to the VWF promotor by binding of transcription activator GATA binding protein 6 (GATA6) [47]. The authors proposed a model in which epigenetic modifications including DNA hypermethylation in non-ECs prevent the association of transcription factors on the VWF promoter. Thus, demethylation is needed to induce binding of transcription activators. Simultaneously, binding of GATA6 further alters its epigenetic landscape by recruitments of PCAF. Bed-specific regulations, based on the location of the EC in the vasculature, may then be achieved by binding of repressors such as NFI or NFY on the already activated promoter.

Noteworthy, non-ECs can gain the ability to express VWF. In particular, some cancer cells are known to activate VWF expression to promote a more malignant phenotype (see Chapter 2.8). This activation was found to rely on epigenetic modifications of the VWF gene promotor, as investigated in tumor cell lines by Mojiri et al. Several studies on the VWF-expressing osteosarcoma cell line Saos2 and glioma cell lines displayed a transcription factor binding pattern similar to the pattern found in active VWF promoters of ECs. Moreover, histone acetylation and DNA demethylation were observed in these VWF-expressing cell lines, indicating that epigenetic modifications can render the activation of VWF expression [49,50]. 

#### 2.1.2. Regulation of Weibel–Palade Body Synthesis and Angiogenesis

The vascular endothelium is a monolayer of ECs forming a barrier between the blood and tissue which controls the passage of molecules and cells. Furthermore, it regulates several physiological processes such as hemostasis and angiogenesis by the secretion of soluble factors. As mentioned above, expression of VWF and the formation of WPBs are characteristic for ECs and ensure the availability and accessibility of VWF upon injury [51]. WPBs are rod-shaped storage organelles formed in the trans-Golgi network (TGN) at a pH of 6.2 [52]. There, ultra-large multimers of VWF assemble into hollow, fenestrated, right-handed helical tubules with repetitive units. 

Impaired formation of WPBs was observed for several VWF mutants, for example, p.Asn528Ser in the D2 domain, which causes absence of HMWM and abolishes the formation of WPBs [53]. Patients harboring this mutation did not respond to desmopressin (DDVAP) treatment, which induces the secretion of the total VWF protein from ECs. Immunofluorescence staining of HEK293, AtT-20 and aortic ECs from type 3 VWD dogs expressing p.Asn528Ser revealed a retention of this mutant in the ER, indicating a storage defect. Similar results were found for a deletion of aa 437–442 in the D2 domain [54], for p.Arg273Trp in the D1 domain [55] and for p.Cys1157Phe and p.Cys1234Trp in D’D3 [56], pointing out that essential information for the formation of WPBs is encoded in domains D1 to D3. Accordingly, deletion of the pro-peptide’s D1 or D2 domain resulted in defective VWF multimerization and storage [57]. Further studies by Huang et al. confirmed that shortened N-terminal VWF fragments containing only D1 to D’D3 are sufficient to initiate packaging of helical tubules and formation of WPBs [52]. 

In some cell types (e.g., HEK293 cells) which normally do not express VWF and are void of WPBs, recombinant expression of VWF can initiate the formation of so-called “pseudo-WPBs” [58,59]. This indicates that all information for the tubule formation and creation of WPBs is annotated in the VWF protein. 

WPBs serve as storage organelles not only for VWF but also for a number of additional proteins, including cytokines and mediators of hemostasis, angiogenesis and inflammation. Known cargo proteins are, for example, P-selectin (P-sel), interleukin-8 (IL-8), angiopoietin-2 (Ang-2), osteoprotegerin (OPG), endothelin-1, endothelin-converting enzyme and calcitonin gene-related peptide [60]. Some of these proteins, such as P-selectin or Ang-2, are targeted to WPBs by direct binding to VWF [61]. Accordingly, VWF serves as a shuttle, sorting proteins into these secretory organelles, and it is likely that defects in WPB formation due to altered VWF expression may perturb their correct distribution. For example, it was shown that WPB localization of Ang-2, an essential regulator of angiogenesis and inflammation, is VWF-dependent as loss of VWF dysregulates its storage [61]. Due to this observation, and the fact that patients with VWD can suffer from angiodysplasia, Starke et al. postulated a role of VWF in angiogenesis [62]. When they isolated blood outgrowth endothelial cells (BOECs) from VWD patients, they found increased angiogenesis of these cells. This finding supports the hypothesis that angiodysplasia in these patients is a consequence of reduced levels of VWF in ECs and thus increased angiogenesis. Extracellular VWF was unable to rescue the VWF knockdown phenotype which may explain the persistence of angiodysplasia in patients treated by plasmatic VWF substitution [62].

The authors further treated ECs with VWF siRNA and observed increased cell migration, while directionality in the cells’ movement was decreased. Moreover, in these experiments, addition of extracellular VWF did not reverse the phenotype, showing that intracellular VWF is essential for this regulatory pathway. However, the observed increased migration of VWF knockdown cells was restored to normal using an inhibitor for the vascular endothelial growth factor (VEGF) receptor, indicating that the role of intracellular VWF in angiogenesis involves VEGF signaling. 

The integrin αvβ3 is the best-characterized endothelial receptor for VWF. Since αvβ3 was described to play multiple roles in angiogenesis and neovascularization [62,63], Starke et al. further investigated if the observed phenotype of VWF-deficient cells might be linked to αvβ3. Indeed, downregulation of VWF expression by siRNA further reduced the surface expression of the αvβ3 integrin and enhanced its internalization [62]. These effects were postulated to be mediated by VWF’s RGD motif since intracellular trafficking of αvβ3 was recently shown to be affected by RGD mimetics [64]. 

**Figure 2:**
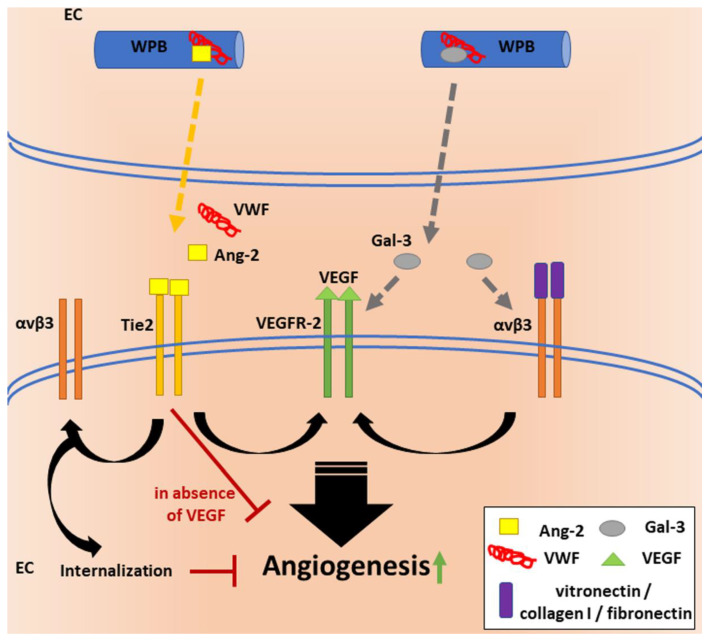
VWF’s regulatory functions in angiogenesis.VWF is essential for the formation of WPBs in endothelial cells (ECs) and regulates the storage and release of cargo proteins, such as growth factor Ang-2. Release of Ang-2 and subsequent binding to its receptor Tie-2 can destabilize blood vessels and promote angiogenesis cooperatively with VEGF signaling through VEGFR-2. In contrast, Tie-2 signaling has anti-angiogenic effects in the absence of VEGF. Ligand binding (e.g., vitronectin, fibronectin, collagen I) to αvβ3 has been shown to increase VEGFR-2 activity, to initiate downstream signaling and to thereby increase EC proliferation. Another protein stored in WPBs is the glycan-binding protein Gal-3. Gal-3 promotes angiogenesis through pathways that involve both αvβ3 and VEGFR-2. Thus, VWF may regulate angiogenesis at multiple levels. Intracellularly, it has anti-angiogenic effects when regulating the storage of proangiogenic factors, but it might also function as a proangiogenic extracellular ligand.

Therefore, what is the link between VWF, VEGF signaling, Ang-2 and αvβ3? VWF seems to regulate angiogenesis by multiple pathways (Figure 2), most likely, primarily, through its shuttle function, guiding cargo proteins to WPBs and regulating their release. In the absence of VWF, formation of WPBs is disturbed, leading to mislocalization of their cargo proteins and an uncontrolled secretion. Thus, VWF knockdown increases extracellular levels of Ang-2. Thomas et al. found that incubation of HUVECs with extracellular Ang-2 leads to internalization and lysosomal degradation of the αvβ3 integrin [65], which suggests that reduced levels of VWF lead to decreased surface exposure of αvβ3 in an Ang-2-dependent manner. These data indicate that VWF is involved in the regulation of Ang-2 signaling by guarding its spatiotemporal intra- and extracellular distribution.

The impact of αvβ3 on angiogenesis was investigated by seeding ECs on αvβ3 binding ligands, such as fibronectin, collagen I and vitronectin, which induced VEGF signaling and a resulting increased EC proliferation [66]. Whether binding to VWF has similar effects was not investigated. Nonetheless, it is surprising that Starke et al. observed increased angiogenesis upon enhanced internalization and thus decreased surface expression of αvβ3. These data suggest that alternative pathways are involved in this complex process. Data from Maisonpierre et al. indicate that regulation of the receptor Tie-2 by its ligands Ang-1 and Ang-2 may have positive or negative downstream effects depending on the combination of simultaneously acting angiogenic signals. Ang-1 may provide signals for maturation or stabilization of the vessel wall via Tie-2, which can be blocked by binding of Ang-2 to Tie-2, resulting in continued remodeling or the initiation of vascular sprouting in the presence of VEGF. Visa versa, Ang-2 binding could result in vessel regression in the absence of VEGF [67], which could explain the anti-angiogenic effect of VEGFR inhibitors and indicate proangiogenic effects through the Ang-2/Tie-2 axis upon reduced αvβ3 surface expression, as observed by Starke et al. [62]. 

Another ligand which can activate the proangiogenic effects of VEGFR, by activation of αvβ3, is the glycan-binding protein galectin-3 (Gal-3) [68]. Similar to Ang-2, Gal-3 also happens to be a WPB-localized molecule that was shown to directly bind to VWF intra- as well as extracellularly [69]. Therefore, Gal-3 might also be involved in VWF-regulated angiogenic processes.

These (patho)physiologically highly relevant findings require further investigation to gain deeper insights into the underlying signaling events and potential direct effects of VWF on the different steps of new vessel formation.

### 2.2. VWF and Vascular Smooth Muscle Cells (VSMCs)

Vascular smooth muscle cells (VSMCs) are located in the tunica media which embraces the tunica intima composed of the endothelium and the extracellular matrix of a vessel. They assure structural integrity and regulate the blood pressure by contraction and relaxation. Additionally, VSMCs have been reported to be involved in the development of atherosclerosis and plaque angiogenesis. Cardiovascular risk factors can activate ECs, triggering a cascade of events, including the recruitment of leukocytes. Further, a highly mitogenic environment is generated by the release of cytokines and growth factors by inflammatory and vascular cells. VSMCs migrate, proliferate and synthesize extracellular matrix components on the luminal side of the vessel wall. These events then allow the formation of the fibrous cap of the atherosclerotic lesion. Thinning of the fibrous cap is subsequently achieved by expression of proteases in response to inflammatory mediators, turning the plaque into a “Jack in the box”, and consequently leading to rupture and thrombus formation. In an advanced disease stage, fibroblasts and VSMCs with extracellular calcification finally cause fibrocalcific lesions [70].

The first implications that deposition of VWF in the subendothelium could play a role in these vascular pathologies mediated by VSMCs were found about two decades ago. Kockx et al. showed that vascular surgery by placement of a flexible silicone cuff around rabbit carotid arteries induced the formation of neointima, which is composed of smooth muscle cells (SMCs). During this process, VWF expression increased in the surrounding ECs, and VWF was deposited in the extracellular spaces of the neointima within 14 days. Later on, VWF deposits disappeared from the neointimal matrix. The physiological role of VWF in the subendothelium was postulated to increase the adhesiveness of the extracellular matrix for ECs and to be a modulator of neointima formation [71]. The latter is essential to wound healing and reconstruction of the damaged vessel. The same group further found VWF localized around SMCs in cholesterol-induced atherosclerotic plaques in rabbits [72]. Additionally, balloon angioplasty of rabbit carotid arteries as well as of porcine carotid arteries was found to be associated with deposition of VWF and intimal thickening [73,74]. 

Neointimal hyperplasia refers to the proliferation and migration of VSMCs primarily in the tunica intima. This process then results in the thickening of arterial walls and a decreased arterial lumen space primarily in response to surgery. Qin et al. found VWF in neointimal hyperplasia of vascular grafts in a rat model [75]. These data led them to investigate whether VWF also has mitogenic effects on mouse SMC proliferation [76]. Indeed, VWF enhanced SMC growth when added to the culture medium in a dose-dependent manner. For in vivo investigations, the authors used a murine model of intimal hyperplasia (IH), which was induced by low shear stress. In this setting, IH was proportional to VWF expression. 

Scheppke et al. investigated the correlation between blood vessel maturation, which involves the coverage of nascent vessels by VSMCs, and the Notch signaling pathway in mice. They found that pharmacological inhibition of Notch signaling led to a reduction in the arterial length coated by VSMCs and increased areas of incomplete VSMC coverage. By observing the development of retinas in a mouse model void of the endothelial Notch ligand Jag1, this very ligand was identified to be a regulator of arterial maturation. Jag1-mediated Notch signaling induces expression of integrin αvβ3 on arterial-associated VSMCs. This is where VWF comes into play, as it has previously been reported that VWF binds to ECs by interaction of its RGD motif-containing C4 domain with integrin αvβ3 on the EC surface [77]. They used two approaches to block this interaction: (1) employing an inhibitory antibody binding to the RGD motif of VWF, and (2) addition of an antibody blocking αvβ3. Both approaches did reduce the adhesion of VSMCs to VWF. Based on their data, the authors suggested the following new roles for Notch and VWF in the regulation of arterial maturation: First, ECs display Jag1 that binds to Notch on VSMCs. Second, this interaction leads to upregulation of αvβ3 expression on VSMCs which enables binding of VWF. Thus, arteries employ Notch signaling between ECs and local perivascular cells to increase integrin αvβ3 expression on VSMCs to facilitate interaction with VWF in the endothelial basement membrane and potentiate the maturation of newly formed arteries [78]. 

Recently, a comprehensive study by Lagrange et al. revealed the specific receptors and signaling pathways by which VWF induces VSMC proliferation in humans [79]. First, they confirmed the link between VWF and VSMC proliferation observed by Qin et al. by comparing wildtype and VWF knockout mice in two distinct models of vascular injury: (1) ligation of the common carotid artery, and (2) femoral artery denudation. Then, VWF was proven to be localized also in the intima and core of human atherosclerotic lesions. To determine the underlying molecular pathway, primary human VSMCs were subjected to a number of experiments showing that VWF has the potential to stimulate migration and proliferation, similar to the known mitogenic protein platelet-derived growth factor composed of two B subunits (PDGF-BB). The VWF-activated signaling pathway was identified by protein phosphorylation studies revealing that VWF significantly induced phosphorylation of p38-MAPK and Src followed downstream by Akt and ERK1/2 phosphorylation. Similar to Scheppke et al., Lagrange et al. observed an involvement of αvβ3 in VWF-induced VSMC proliferation because knockdown of αv by siRNA annihilated the proliferative effect of VWF on VSMCs. This treatment further inhibited the VWF-dependent Src phosphorylation. Surprisingly, addition of either a cRGDPV peptide or an anti-αvβ3 integrin antibody, both of which would block the binding of the VWF-RGD motif to αvβ3, did not prevent VWF-mediated VSMC proliferation. These data indicate that VWF-dependent activation of VSMC proliferation via the MAPK/Erk pathway requires a co-receptor, in contrast to the Notch pathway, which was shown to function by direct interaction of VWF with αvβ3 [78]. Indeed, the authors were able to identify the LDL-related receptor protein (LRP) 4 as the direct binding partner of VWF which associates with the A2 domain of VWF instead of the C4 domain [79]. 

### 2.3. VWF and Platelets

#### 2.3.1. VWF in Platelet Formation and Morphology

Megakaryocytes (MKs) are the mother cells of platelets. They are large, granular and polyploid cells with a size of 60 µm in diameter, which derive from hematopoietic stem cells in the bone marrow [80]. They are mainly found in the perivasculature, in close proximity to sinusoidal endothelial cells. Their process of maturation is called megakaryocytopoiesis and is characterized by an increase in cell size and ploidy as well as enhanced synthesis of storage organelles and invaginated plasma membranes. This process is primarily induced by the cytokine thrombopoietin (TPO), which is constitutively expressed by the liver [81]. In the later stages of megakaryocytopoiesis, MKs will reorganize their cytoskeleton to form pseudopodal elongations, so-called proplatelets, from which platelets will be separated [82]. During this process, protrusions extend through the endothelium to release platelets into the blood stream, while the main MK remains perivascular. 

Interestingly, Balduini et al. described that MK interaction with VWF initiates proplatelet formation within 16 h [83] which is accelerated to 20 min when MKs are subjected to high shear forces [84]. Similar to the process of platelet adherence to VWF, this binding was shown to be based on the interaction of the VWF A1 domain with GPIbα expressed on the MK cell surface. This was underlined by the fact that the presence of a GPIbα-inhibiting antibody completely abolished proplatelet formation and platelet release. Equally, in patients with idiopathic thrombocytopenic purpura (ITP) who develop antibodies against GPIbα, impaired MK maturation and platelet production were also observed [85,86]. Blocking antibodies against GPIIb/IIIa further showed the crucial involvement of this receptor in proplatelet elongation and platelet formation under flow conditions [84]. Conversely, Nurden et al. observed that tirofiban, which inhibits GPIIb/IIIa, did not block the VWF-induced enhancement of platelet production under static conditions [87].

Similar effects of intracellular VWF in MKs on platelet production were observed by Nurden et al., who further investigated the importance of the VWF–GPIbα interaction in megakaryocytopoiesis. Patients with VWD type 2B carry mutations in the VWF A1 domain that lead to enhanced GPIbα binding of VWF. Counterintuitively, this effect induces bleeding symptoms because platelets spontaneously bind to VWF, resulting in the formation of circulating VWF–platelet complexes in some patients as well as increased clearance of VWF and platelets. The authors found platelets from VWD2B patients to display non-spherical shapes with an altered membrane and granule distribution. Some platelets seemed to be fused with the membrane of other thrombocytes, and the formation of giant or enlarged platelets was observed in all affected individuals to different extents [87]. These data indicate a direct role of intracellular VWF in MKs in megakaryocytopoiesis.

#### 2.3.2. VWF in Platelet Signaling and Apoptosis

The most prominent cellular function of VWF is the activation of platelets during primary hemostasis. This process requires binding of platelets to immobilized VWF, which first leads to reversible adhesion of platelets and later to stable aggregation and clot formation. While the basic function and binding interactions of platelets are well studied, novel aspects of more complex and ramified signal transduction pathways are continuously discovered in platelets (reviewed in [88]). In principle, platelet activation is characterized by activation of phospholipase C and a rise in the intracellular calcium ion (Ca^2+^) concentration [89], which promotes GPIIb/IIIa activation, cytoskeletal rearrangement and granule secretion [90,91,92]. Activation of platelets can be promoted by various soluble factors via binding of different receptors such as collagen via the glycoprotein GPVI [93], fibrinogen via GPIIb/IIIa [94], LPS via Toll-like receptors [95] or thrombin via GPIb-IX or PAR receptors [96,97]. In contrast, VWF activates platelets via the GPIb-IX-V signaling pathway [98]. Shear force-mediated activation of VWF unfolds the A1 domain and enables binding to the GPIb-IX-V complex, in particular, the GPIbα subunit [99]. Binding to GPIbα triggers a conformational change of the mechanosensitive domain (MSD) within the extracellular region of GPIbα [100]. The same mechanism was also found for the leucine-rich repeat domain (LRRD) in GPIbα [101]. The mechanical stretching of these domains is converted into intracellular signal transduction by the intracellular adapter protein 14-3-3ζ [102,103]. These regulatory mechanisms based on mechanical forces assure the activation of platelets only when it is needed. Further intracellular signaling pathways involve the association of phosphoinositide 3-kinase (PI3K) and Src family protein kinase (SFK) members [104,105]. Activation of PI3K leads to generation of phosphatidylinositol-(3,4,5)-trisphosphate (PIP_3_), which mediates the membrane translocation of 3-phosphoinositide-dependent kinase 1 (PDK1) and Akt isoforms, allowing the phosphorylation and activation of Akt [106]. The PI3K-Akt signaling pathway further activates downstream effectors, e.g., nitric oxide synthase (NOS) [107]. NOS is activated by VWF binding to GPIb-IX, which stimulates Ca^2+^ mobilization, the activity of Src family kinases, PI3K and phospholipase C (PLC), resulting in the formation of cGMP. Downstream sequential activation of the cGMP-dependent protein kinase G (cGMP-PKG), p38 and the mitogen-activated protein kinase (MAPK)-extracellular signal kinase (ERK) pathway ultimately leads to activation of integrin αIIbβ3 [108,109]. This GPIb-dependent stimulating pathway is distinct from a GPIb-independent pathway, which leads to inhibition of platelet function through synthesis of cGMP. Here, endothelial prostacyclin and NO initiate the production of cGMP by stimulating GMP cyclases and subsequent activation of cGMP-PKG, which then phosphorylates multiple substrates involved in platelet inhibition (reviewed in [110]).

The interaction of VWF and GPIbα is reversible and corresponds to platelet adhesion, whereas stable aggregation of platelets requires the activation of the GPIIb/IIIa receptor built by integrins αIIbβ3 [111]. In resting platelets, GPIIb/IIIa is inactive [88], and its activation requires an intracellular impulse, so-called inside-out signaling, which is mediated by the MAPK-p38-Erk signal pathway [108,109] and subsequent association of Talin 1 to the cytoplasmic domains of GPIIb/IIIa. This results in a conformational change of the extracellular domains, which enhances the affinity for agonist binding. Proteins with an integrin binding site can bind activated GPIIb/IIIa on platelets and initiate outside-in signaling, resulting in stable aggregation. The latter is achieved by Syk-mediated activation of PLC-Y2, which catalyzes the hydrolysis of PIP_2_ into diacylglycerol (DAG) and inositol-(1,4,5)-trisphosphate (IP3) [112]. IP3 binds to the IP3 receptor in the ER membrane, leading to release of Ca^2+^ from the dense tubular system into the cytosol [89]. This further stimulates store-operated calcium entry (SOCE), elevating the intracellular Ca^2+^ concentration [113]. Calcium plays a central role in platelet activation as it is required for nearly all functions including stable platelet adhesion, aggregation, cytoskeleton rearrangement and secretion of granular content. 

The altered interaction of GPIIb/IIIa with VWF via its RGD motif in the C4 domain [114] was recently identified to be pathophysiologically relevant. In an interdisciplinary study, we and our partners identified the single-nucleotide polymorphism p.Phe2561Tyr within C4 to be the first prothrombotic VWF variant. The amino acid exchange leads to destabilization of the stem region that reduces the critical shear rate for VWF-GPIIb/IIIa interaction, thereby promoting platelet aggregation at lower shear rates and thus increasing the risk of myocardial infarction [30]. 

Besides their activation, apoptosis of platelets was also found to be induced by VWF [115]. Likewise, this process is stimulated by interaction of VWF with GPIbα on the platelets’ surface. Li et al. reported that Ristocetin-induced VWF–GPIbα interaction activates mitochondria-dependent platelet apoptosis by activation of the pro-apoptotic regulators Bax and Bak, cleavage of gelsolin and depolarization of ΔΨ_m_. This process is independent of platelet activation as enhanced apoptosis can still be observed after incubation with platelet inhibitors PGE_1_ and apyrase.

### 2.4. Cells Involved in VWF Clearance

#### 2.4.1. Macrophages

Van Schooten et al. investigated the clearance of human VWF injected in VWF-deficient mice and recovered most of the protein from liver and spleen tissue, where it was targeted to macrophages. In vitro, binding, uptake and degradation of VWF were confirmed in human macrophages by immunofluorescence studies. Alongside VWF, FVIII is also found in macrophages, which shows that VWF-bound FVIII is cleared via the same pathway [116]. In 2012, two studies provided deeper mechanistic insights into the degradation of the VWF–FVIII complex [117,118]. Both studies provided strong evidence that in mice and humans, low-density lipoprotein (LDL) receptor-related protein-1 (LRP1) mediates the uptake of the VWF–FVIII complex into macrophages in a shear stress-dependent manner [117], and with a contribution of β2 integrins [118]. Since binding of VWF to macrophages was also observed under static conditions [116], Wohner et al. searched for additional receptors. As VWF plasma levels were shown to be associated with the epithelial cell-specific receptor SCARA5 in genome-wide association studies (GWAS) [119], they investigated the related macrophage scavenger receptor SR-AI as a putative candidate. Indeed, VWF was able to bind to SR-IA under static conditions in a calcium-dependent manner. The VWF binding domains for SR-IA are located in the D’D3 and the D4 domains, and the authors suggested that the same domains are also involved in LRP1 interaction [120]. 

VWF and FVIII are highly sialylated proteins and could thus be ligands for sialic acid-binding immunoglobulin-like lectins (Siglecs). By performing binding experiments with purified VWF and FVIII as well as cell binding studies with Siglec-5-expressing cells, Pegon et al. showed that VWF and FVIII are ligands for Siglec-5, and binding results in their endocytosis [121]. Thus, Siglec-5 seems to be involved in the regulation of VWF–FVIII plasma levels. Furthermore, desialylation has been described as a general marker for protein clearance. Removal of sialic acid residues exposes galactose (Gal) or N-acetylgalactosamine (GalNAc) on glycan branches, which are ligands of specific clearance receptors. Hyposialylated VWF is thus also recognized for clearance by the Ashwell–Morrell receptor [122] as well as the macrophage galactose-type lectin (MGL) receptor [123]. 

#### 2.4.2. Sinusoidal Endothelial Cells (LSECs) 

Von Willebrand disease type 1 patients exhibit decreased plasma levels of VWF, but in some patients no causative mutation can be identified in the VWF gene itself. As a novel candidate influencing the VWF half-life in the circulation, a single-nucleotide polymorphism (SNP) within or near the C-type lectin receptor, C-type lectin member 4 family M (CLEC4M), showed a consistent statistical association with VWF and FVIII levels in a GWAS [119]. Employing immunofluorescence, ELISA and FACS studies, Rydz et al. were able to show that CLEC4M, a calcium-dependent mannose-specific receptor, mediates binding and internalization of VWF into CLEC4M-expressing HEK cells depending on the glycosylation status of VWF. Since CLEC4M is predominantly expressed in sinusoidal endothelial cells (LSECs), these cells are assumed to be the major source of CLEC4M-mediated clearance in the liver and spleen [124]. Another locus identified in the GWAS meta-analysis, namely, stabilin-2, was investigated later, and also for this receptor, a role in VWF clearance could be shown in LSECs [125]. 

### 2.5. VWF and Immune Cells

#### 2.5.1. VWF and Leukocytes

When leukocytes invade inflamed tissue, three steps are important: rolling and adhesion to the endothelium and extravasation through the EC layer.

Pendu et al. demonstrated that the VWF protein itself provides binding sites for a subset of leukocytes, such as monocytes and polymorphonuclear leukocytes (PMNs). In their experiments, P-selectin glycoprotein ligand-1 (PSGL-1) was shown to be a VWF binding ligand involved in leukocyte rolling and the leukocyte-specific β2-integrin family-mediated stable leukocyte adhesion via VWF interaction [126]. In contrast, Petri et al. found rolling and adhesion to be unaffected when VWF-blocking antibodies were present in a thioglycollate-induced peritonitis model in mice. However, plasma-derived VWF was essential for leukocyte passage through the EC layer as anti-VWF antibodies reduced extravasation by about 50%. This effect was blocked by inhibitory antibodies against GPIbα, indicating that VWF supports leukocyte extravasation by recruiting platelets via GPIbα [127]. This hypothesis was later supported by Aymé et al., who showed that an antibody specifically blocking the GPIbα-binding A1 domain fully inhibited the vascular permeability in the two mouse models used in this study as the observed leakage of hemoglobin was reduced to background levels. Additionally, leukocyte recruitment was almost completely lost in the irritant contact dermatitis model and the immune complex-mediated vasculitis (ICV) model [128]. In contrast to the peritonitis model, Hillgruber et al. found VWF to be involved in leukocyte recruitment to the skin in cutaneous inflammation models, suggesting that the mechanisms leading to leukocyte recruitment are heterogeneous and critically depend on the type of inflamed tissue [129,130].

Petri et al. further suggested that platelets bound to VWF and presented on the endothelium of inflamed mesenteric venules promote the opening of endothelial junctions, thereby facilitating the transmigration process because, in their experiments, VWF-blocking antibodies also reduced vascular permeability [127]. During this diapedesis process, actual plasma leakage needs to be prevented. Braun et al. recently suggested that VWF is also involved in this process. They found that platelets recruited to the site of injury by VWF release Ang-1, which then activates the receptor tyrosine kinase Tie-2 and its downstream target FGD5 in ECs [131]. FGD5 (FYVE, RhoGEF and PH domain-containing protein 5) is a member of the Ras-like family of Rho and Rac proteins that acts as a Cdc42 guanine nucleotide exchange factor (GEF), which was previously shown to be required for Tie-2-stimulated Cdc42 and Rac1 activation, supporting the reinforcement of cortical actin and dampening of radial stress fibers [132]. Therefore, they suggested that the Tie-2–FGD5 axis is essential to promote the tightening of junctions by strengthening actin bundles during leukocyte transmigration through the diapedesis pore [131].

Apart from the above-described direct roles of VWF in inflammation, additional indirect roles have been suggested (reviewed in [133]) as VWF regulates the storage and secretion of P-selectin (P-sel), which is a known player in inflammation. It is commonly accepted that leukocyte rolling is initiated by binding of P-selectin glycoprotein ligand-1 (PSGL-1) to P-sel on the EC surface. This is especially important when immune cells need to pass the endothelium to reach a point of inflammation [134]. P-sel storage in WPBs is guided by VWF through binding of P-sel to the VWF D’D3 domain [135]. Studies of VWF-deficient mice revealed impaired storage and disrupted exposure of P-sel at the cell surface of ECs. Consequently, these defects result in reduced adhesion and recruitment of leukocytes [136]. Whether there is also a clinical impact of diminished P-sel exposure in patients with VWD is not entirely clear yet. One study investigated endothelial function in subjects with VWD types 1, 2 and 3 and found higher plasma levels of P-sel, but the difference did not reach statistical significance [137]. 

Bernardo et al. investigated string-like structured UL-VWF molecules released from ECs that are decorated by platelets upon GPIbα binding. These platelets become activated and expose P-sel, which supports leukocyte rolling, even under the high shear stresses found in arteries and arterioles [138].

Furthermore, high levels of VWF in response to endothelial activation have been shown for a variety of inflammation-related pathologies, as also shown recently for COVID-19 [139]. However, data on the reliability of VWF as a marker of inflammatory response and disease outcome are not consistent in humans yet [133,140]. Nonetheless, the interactions of VWF with cells of the immune system suggest a supportive role of VWF in inflammation. This is underlined by the fact that inflammatory stimuli, for example, inflammatory cytokines such as tumor-necrosis factor (TNF) and interleukin-1 (IL-1), can stimulate and activate the VWF-expressing ECs and induce secretion of their WPB content [141]. As VWF is the main cargo of WPB, it thus represents a marker of inflammation and an acute phase response protein [133]. 

#### 2.5.2. VWF and Neutrophils

Neutrophils expose the Slc44a2 protein on their cell surface which was shown to be a VWF receptor. Zirka et al. recently uncovered that expression of the human neutrophil antigen 3b (HNA-3a) is required for Slc44a2-mediated neutrophil adhesion to VWF at venous shear rates. This interaction was increased by pre-activation of neutrophils with lipopolysaccharide and independent of the β2 integrin. Slc44a2 knockout mice further exhibited a strong reduction in neutrophil recruitment in inflamed mesenteric venules [142]. This VWF-mediated recruitment of neutrophils could play a role in venous thromboinflammation. 

Additionally, VWF was found to directly bind to DNA in neutrophil extracellular traps (NETs) [143] via specific arginine residues in its A1 domain [144], indicating a role of VWF in sepsis and pathogen defense. Moreover, VWF can also bind directly to histones of NETs [145]. VWF included in NETs was also found to promote leukocyte recruitment, contributing to an enhanced inflammatory response [146].

#### 2.5.3. VWF and Dendritic Cells

The development of alloantibodies against FVIII in patients with hemophilia A is a common problem, and it has been suggested that VWF present in FVIII concentrates may protect FVIII not only from clearance but also from internalization by dendritic cells (DCs). In vitro, it was confirmed that DCs internalize FVIII dose-dependently, leading to the presentation of FVIII peptides and subsequent activation of the anti-FVIII C1 domain-specific human CD4+ T cell clone D9E9 [147]. Preincubation of FITC-labeled FVIII with VWF indeed decreased the internalization of FVIII in DCs, and the resulting activation of D9E9 was significantly reduced. Whether this protective effect is also physiologically relevant remains to be confirmed [147]. 

### 2.6. VWF and Osteoclasts

Osteoprotegerin (OPG) is a member of the TNF receptor family, which can act as a negative regulator of osteoclastogenesis [148]. Employing immunofluorescence, VWF and OPG were found to co-localize in WPBs of HUVECs [149,150] and α-granules in platelets [151]. Stimulation of ECs leads to secretion of both proteins. Co-immunoprecipitation experiments showed complex formation of VWF and OPG within ECs as well as in the cell medium after cytokine-induced secretion [150]. Binding of VWF to OPG is calcium-dependent, and the interaction displays strong pH dependence, with optimal binding occurring at pH 6.5, the pH of the Golgi apparatus, indicating that OPG is associated with VWF throughout intracellular trafficking. The presence of VWF in complex with FVIII does not alter the interaction with OPG, and FVIII itself is unable to bind to OPG [149]. This finding can be explained by the independent binding sites of OPG and FVIII in the VWF A1 and D’D3 domains, respectively. 

**Figure 3:**
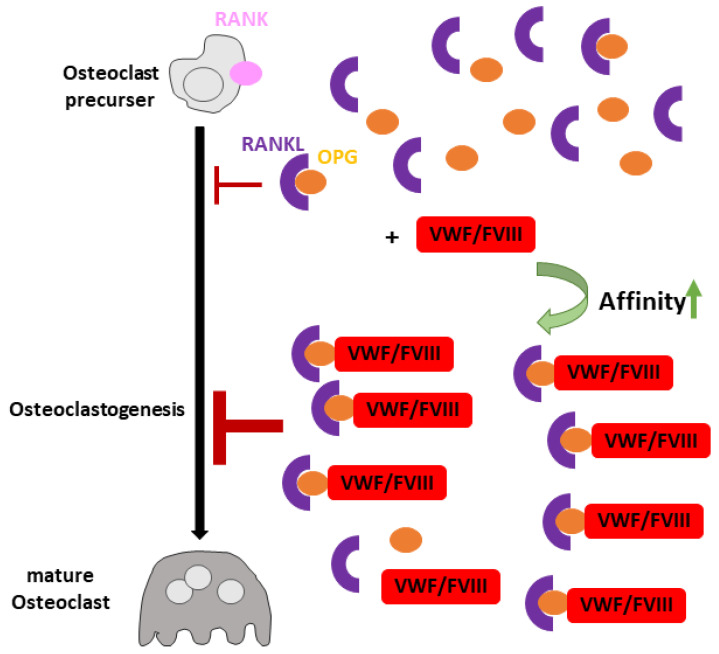
VWF’s role in osteoclastogenesis.In the absence of the VWF–FVIII complex (red), OPG (orange) binds to Receptor Activator of Nuclear factor κB Ligand (RANKL, violet), leading to inhibition of osteoclastogenesis by preventing RANKL from binding to its receptor RANK (pink). In the presence of the VWF–FVIII complex, the affinity of OPG to RANKL is further increased, enhancing the inhibitory effect of OPG on osteoclastogenesis.

To fulfill its role as an inhibitor of osteoclastogenesis [152,153], OPG acts as a decoy receptor for Receptor Activator of Nuclear factor κB Ligand (RANKL). This interaction prevents the binding of RANKL to its receptor RANK, which induces osteoclastic differentiation and maturation, leading to bone resorption [154,155]. Baud’huin et al. investigated the contribution of VWF in these processes and found only the VWF–FVIII complex to increase the inhibitory effect of OPG on RANKL-induced osteoclastogenesis by an additional 18%. FVIII alone had no effect. Surface plasmon resonance measurements showed that the VWF–FVIII complex interaction with OPG increased the binding affinity of OPG to RANKL, thereby enhancing the inhibitory effect of RANKL on osteoclastic differentiation [156] (Figure 3). 

### 2.7. VWF and Cancer Cells

**Figure 4:**
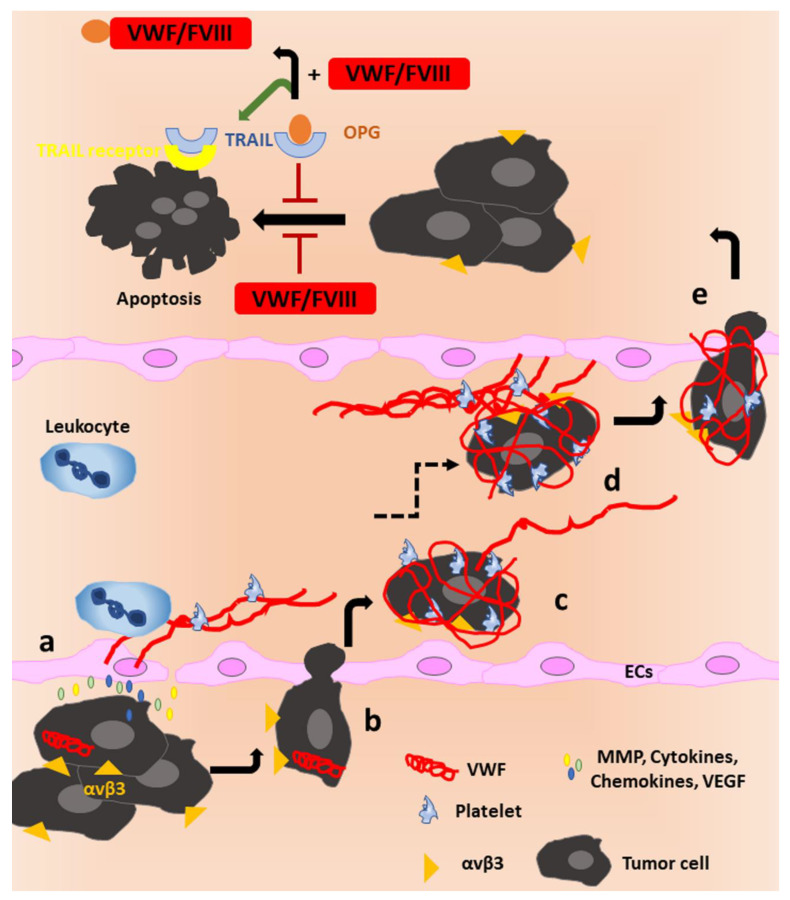
VWF’s role in tumor cell apoptosis and metastasis.Upper part: In the absence of the VWF–FVIII complex (red), OPG (orange) binds to Tumor necrosis factor-Related Apoptosis-Inducing Ligand (TRAIL, blue), leading to inhibition of tumor cell apoptosis. The presence of the VWF–FVIII complex functions as a decoy receptor for OPG, and TRAIL is released to interact with its receptor, inducing apoptosis of the tumor cell. If only VWF is present, it has anti-apoptotic properties, promoting tumor cell survival. Lower part: **a**: Tumor cells release a variety of proteins (matrix metalloproteinases (MMPs), vascular endothelial growth factor (VEGF), cytokines and chemokines) to induce disintegration of the subendothelial extracellular matrix and to activate the endothelium. ECs then release ultra-large (UL-)VWF, increasing the VWF antigen and promoting the formation of platelet-decorated strings as well as recruitment of leukocytes. **b**: The resulting proinflammatory cytokine signaling, enhanced angiogenesis, and altered vascular permeability create a premetastatic environment, promoting tumor growth and the transmigration of tumor cells through the endothelium into the blood stream. **c**: The metastatic cells can bind VWF via integrin αvβ3 and shield themselves from detection by immune cells by cloaking themselves in a shell of VWF and platelets. Some tumor cells acquire the ability to produce VWF to further potentiate these effects. **d**: Interaction of tumor cells with VWF and platelets may further support adhesion to the vessel wall, helping the cells to initiate. **e**: extravasation to the metastatic site.

OPG is also an inhibitor of Tumor necrosis factor-Related Apoptosis-Inducing Ligand (TRAIL) [157], a pro-apoptotic cytokine inducing cancer cell death [158]. The presence of OPG was shown to inhibit the induction of apoptosis by TRAIL in the human osteosarcoma cell line MG63OPG as OPG binds TRAIL and hinders its cytokine function. In contrast, complex formation of OPG and VWF–FVIII prevents OPG from interacting with TRAIL, thereby promoting the pro-apoptotic function of TRAIL by allowing ligand binding to its receptor [156]. Thus, the VWF–FVIII complex can have a pro-apoptotic effect via TRAIL in the presence of OPG (Figure 4, upper part). Surprisingly, the same study described that the addition of the VWF–FVIII complex alone has a direct anti-apoptotic effect on osteosarcoma cells in the absence of OPG [156], which was not further characterized. 

That VWF can have further pro-apoptotic functions was shown by Mochizuki et al. who observed VWF-induced apoptosis in tumor cells with a low expression of the protease ADAM28 (breast cancer cell line MCF-7, renal cell carcinoma line 769P and hepatocellular cell carcinoma line HepG2). Since high ADAM28 expression in cell lines PC-9 and Calu-3 (lung carcinoma), MDA-MB231 (breast cancer) and Caki-2 (renal cell carcinoma) protected these cells against the VWF-associated pro-apoptotic effect, the authors concluded that ADAM28 cleavage of VWF could counteract the pro-apoptotic, and thus anti-metastatic, effect of VWF. This effect was suggested to be mediated via binding of VWF to αvβ3 and subsequent phosphorylation of tumor protein p53 and activation of caspase 3 [159]. An anti-metastatic function of VWF was also observed by Terraube et al., who injected murine melanoma B16-BL6 cells or Lewis lung carcinoma cells in VWF knockout mice and wildtype mice and found a significant increase in the number of pulmonary metastatic foci in the knockout mice. This phenotype could be prevented by co-injection of the cells with recombinant human VWF [160]. 

Contrarily, Feinauer et al. performed in vivo multiphoton laser scanning microscopy to observe the formation of brain metastases after injection of Jimt1 breast cancer cells in mice and identified a pro-metastatic role of VWF. They observed clot formation to be a prerequisite for the extravasation of tumor cells into the brain tissue. A series of inhibition studies convincingly showed that tumor cell-derived tissue factor (TF) initiates thrombin generation which can subsequently lead to VWF secretion from the endothelium. Direct interaction of the cells with platelets was not observed, indicating that clot proteins such as VWF and fibrin play a pivotal role in arresting tumor cells in the clot. Their data further suggested that VWF is also relevant for the growth of micrometastases as anti-VWF treatment resulted in a reduced count of brain metastases and a smaller mean macrometastasis size in both melanoma and breast cancer models. Additionally, tumor cell survival in the perivascular niche was reduced in the breast cancer model in the presence of anti-VWF antibodies. Here again, the VWF–integrin interaction could come into play [161]. Extravasation could be further promoted by neutrophils which were shown to be recruited by VWF fibers (Figure 4a) [127,162].

Plasmatic VWF has further been shown to be a biomarker for tumor progression as VWF levels are increased in cancer patients with tumors of multiple origins (reviewed in [163]). The rise in the VWF antigen in cancer patients has been attributed to tumor-induced activation of the endothelium which promotes cancer cell intravasation into the circulation (Figure 4a,b). In vitro, addition of tumor secretomes of, for example, PC3 prostate cancer cells [164], melanoma cells (MV3, BLM) [165,166], A549 lung adenocarcinoma cells [167] and RT4 urothelial carcinoma cells [168] to ECs was observed to induce WPB secretion and thus release of VWF. In the blood stream, tumor cells can bind VWF and platelets to shield themselves from being detected by immune cells (Figure 4c) and to promote clot formation, as observed by Feinauer et al. [161]. Here, VWF can function as an adhesive anchor for tumor cells to facilitate docking to the endothelium and subsequent extravasation (Figure 4d,e). This process is likely supported by VWF interaction with αvβ3 on the cancer cell surface, as, for example, Pilch et al. showed that melanoma cells can attach to immobilized VWF under venous flow conditions exclusively by interaction with this integrin [169]. Additionally, expression of GPIbα on the surface of breast cancer cells was shown to promote tumor cell spreading through cytoskeleton rearrangement and tumor migration in vitro when exposed to a surface coated with human VWF [170]. 

Recently, more and more studies have shown that tumor cells can also acquire the ability to synthesize VWF de novo. This is surprising, since VWF expression is strictly limited to ECs and megakaryocytes under physiological conditions. The synthesis of large VWF multimers consumes a lot of energy and resources; thus, cancer cells must gain an advantage by producing VWF, which is mostly presumed to involve an increased potential for metastasis. Immunohistochemistry showed that the VWF protein is expressed in osteosarcoma cells in vivo in 13 out of 29 tumor specimens as well as in vitro in SAOS2. In this osteosarcoma cell line, VWF showed the typical pseudo-WPB-like localization [171] which is also induced in HEK293 cells after recombinant expression of VWF. Mojiri et al. further investigated the effects of VWF expression in SAOS2 cells as well as in the glioma cell line M049 and found that VWF biosynthesis significantly increases tumor cell adhesion to an endothelial monolayer and platelets compared to the non-expressing cell line KHOS. Transwell migration assays and ex vivo experiments in chicken embryos further showed that siRNA treatment against VWF reduced the cells’ transmigration activity [49]. 

In the colorectal cancer cell line SW480, immunocytochemistry showed a positive signal for VWF, which, surprisingly, was mainly located in the nucleus. Treatment with anti-VWF antibody decreased the cells’ proliferation, adhesion and migration [172]. 

Furthermore, Western blotting, immunohistochemical analysis of liver biopsies and ELISA from plasma samples revealed a correlation between VWF expression and hepatocellular carcinoma cell (HCC) clinicopathologic staging. Knockdown of VWF by siRNA impaired the invasion and migration of HCC cells in vitro and reduced Signal Transducer and Activator of Transcription 3 (STAT3), matrix metalloproteinase (MMP) 2 and 9 protein expression [173]. 

For gastric adenocarcinoma, it was further shown that cancer cell-derived VWF can enhance metastasis by mediating cancer cell aggregation and interaction with platelets and ECs [174]. 

Mojiri et al. hypothesized that their findings on VWF’s influence on cancer cell survival might explain the contradictory observations of the pro- as well as anti-metastatic and -apoptotic effects of VWF. They suggested that, on the one hand, cancer cells that do not express VWF may be susceptible to the pro-apoptotic effect of VWF. Such cancer cells might exhibit enhanced survival and metastasis in VWF knockout mice. On the other hand, VWF-expressing cancer cells should continue to metastasize in VWF knockout mice as they have no disadvantage in the absence of plasmatic VWF [49].

## 3. Summary

**Figure 5:**
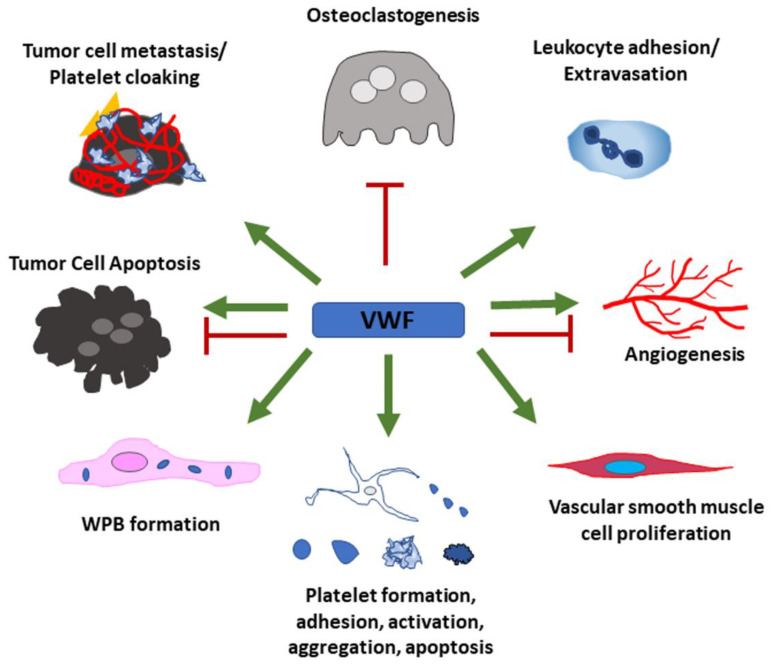
Summary of VWF’s cellular functions.

VWF is a multi-domain, multi-functional protein which can promote a wide range of cellular functions via its different binding sites for a variety of protein binding partners. Figure 5 presents a summarizing overview of the currently known effects of VWF on different cell types that have been discussed in this review. Although these effects have convincingly been shown, a lot of open questions remain as most of the underlying cellular signaling pathways have not yet been entirely uncovered. Further investigation is important as the development of novel therapies targeting VWF has been suggested to help in a broad range of diseases. Some possible goals of these treatment options include, but are not limited to, attenuation of metastasis, reduction in thrombosis, thromboinflammation and atherosclerosis, prevention of myocardial infarction and stroke and intervention in infection and sepsis. 

VWF has positive effects (green arrows) on leukocyte adhesion and extravasation, proliferation of vascular smooth muscle cells and Weibel–Palade body formation in endothelial cells. VWF further regulates every aspect of the platelet life cycle. Tumor cells use VWF to support their metastatic potential and hide from immune cells by cloaking themselves with VWF and platelets. Some tumor cells acquire the ability to produce VWF to further enhance these effects. Depending on the presence of additional effector proteins, VWF can also have pro- as well as anti-apoptotic and -angiogenic properties. Osteoclastogenesis is inhibited by VWF through binding to OPG. 

## References

[1] Sadler J.E. (1998). Biochemistry and genetics of von Willebrand factor. Annu. Rev. Biochem..

[2] Jaffe E.A., Hoyer L.W., Nachman R.L. (1973). Synthesis of antihemophilic factor antigen by cultured human endothelial cells. J. Clin. Investig..

[3] Sporn L.A., Chavin S.I., Marder V.J., Wagner D.D. (1985). Biosynthesis of von Willebrand protein by human megakaryocytes. J. Clin. Investig..

[4] Verweij C.L., Diergaarde P.J., Hart M., Pannekoek H. (1986). Full-length von Willebrand factor (vWF) cDNA encodes a highly repetitive protein considerably larger than the mature vWF subunit. EMBO J..

[5] Sadler J.E., Shelton-Inloes B.B., Sorace J.M., Harlan J.M., Titani K., Davie E.W. (1985). Cloning and characterization of two cDNAs coding for human von Willebrand factor. Proc. Natl. Acad. Sci. USA.

[6] Zhou Y.F., Eng E.T., Zhu J., Lu C., Walz T., Springer T.A. (2012). Sequence and structure relationships within von Willebrand factor. Blood.

[7] Lippok S., Kolsek K., Löf A., Eggert D., Vanderlinden W., Müller J.P., König G., Obser T., Rohrs K., Schneppenheim S. (2016). von Willebrand factor is dimerized by protein disulfide isomerase. Blood.

[8] Zhou Y.F., Eng E.T., Nishida N., Lu C., Walz T., Springer T.A. (2011). A pH-regulated dimeric bouquet in the structure of von Willebrand factor. EMBO J..

[9] Dang L.T., Purvis A.R., Huang R.H., Westfield L.A., Sadler J.E. (2011). Phylogenetic and functional analysis of histidine residues essential for pH-dependent multimerization of von Willebrand factor. J. Biol. Chem..

[10] Purvis A.R., Sadler J.E. (2004). A covalent oxidoreductase intermediate in propeptide-dependent von Willebrand factor multimerization. J. Biol. Chem..

[11] van de Ven W.J., Voorberg J., Fontijn R., Pannekoek H., van den Ouweland A.M., van Duijnhoven H.L., Roebroek A.J., Siezen R.J. (1990). Furin is a subtilisin-like proprotein processing enzyme in higher eukaryotes. Mol. Biol. Rep..

[12] McKinnon T.A., Goode E.C., Birdsey G.M., Nowak A.A., Chan A.C., Lane D.A., Laffan M.A. (2010). Specific N-linked glycosylation sites modulate synthesis and secretion of von Willebrand factor. Blood.

[13] Nowak A.A., Canis K., Riddell A., Laffan M.A., McKinnon T.A. (2012). O-linked glycosylation of von Willebrand factor modulates the interaction with platelet receptor glycoprotein Ib under static and shear stress conditions. Blood.

[14] Solecka B.A., Weise C., Laffan M.A., Kannicht C. (2016). Site-specific analysis of von Willebrand factor O-glycosylation. J. Thromb. Haemost..

[15] Wagner D.D., Mayadas T., Marder V.J. (1986). Initial glycosylation and acidic pH in the Golgi apparatus are required for multimerization of von Willebrand factor. J. Cell Biol..

[16] Carew J.A., Browning P.J., Lynch D.C. (1990). Sulfation of von Willebrand factor. Blood.

[17] Lopes da Silva M., Cutler D.F. (2016). von Willebrand factor multimerization and the polarity of secretory pathways in endothelial cells. Blood.

[18] Hamilton K.K., Sims P.J. (1987). Changes in cytosolic Ca2+ associated with von Willebrand factor release in human endothelial cells exposed to histamine. Study of microcarrier cell monolayers using the fluorescent probe indo-1. J. Clin. Investig..

[19] Levine J.D., Harlan J.M., Harker L.A., Joseph M.L., Counts R.B. (1982). Thrombin-mediated release of factor VIII antigen from human umbilical vein endothelial cells in culture. Blood.

[20] Vischer U.M., Wollheim C.B. (1997). Epinephrine induces von Willebrand factor release from cultured endothelial cells: Involvement of cyclic AMP-dependent signalling in exocytosis. Thromb. Haemost..

[21] Kaufmann J.E., Oksche A., Wollheim C.B., Gunther G., Rosenthal W., Vischer U.M. (2000). Vasopressin-induced von Willebrand factor secretion from endothelial cells involves V2 receptors and cAMP. J. Clin. Investig..

[22] Blair P., Flaumenhaft R. (2009). Platelet alpha-granules: Basic biology and clinical correlates. Blood Rev..

[23] Wagner D.D. (1990). Cell biology of von Willebrand factor. Annu. Rev. Cell Biol..

[24] Romijn R.A., Bouma B., Wuyster W., Gros P., Kroon J., Sixma J.J., Huizinga E.G. (2001). Identification of the collagen-binding site of the von Willebrand factor A3-domain. J. Biol. Chem..

[25] Flood V.H., Schlauderaff A.C., Haberichter S.L., Slobodianuk T.L., Jacobi P.M., Bellissimo D.B., Christopherson P.A., Friedman K.D., Gill J.C., Hoffmann R.G. (2015). Crucial role for the VWF A1 domain in binding to type IV collagen. Blood.

[26] Hoylaerts M.F., Yamamoto H., Nuyts K., Vreys I., Deckmyn H., Vermylen J. (1997). von Willebrand factor binds to native collagen VI primarily via its A1 domain. Biochem. J..

[27] Fu H., Jiang Y., Yang D., Scheiflinger F., Wong W.P., Springer T.A. (2017). Flow-induced elongation of von Willebrand factor precedes tension-dependent activation. Nat. Commun..

[28] Hantgan R.R., Hindriks G., Taylor R.G., Sixma J.J., de Groot P.G. (1990). Glycoprotein Ib, von Willebrand factor, and glycoprotein IIb:IIIa are all involved in platelet adhesion to fibrin in flowing whole blood. Blood.

[29] Sadler J.E., Budde U., Eikenboom J.C., Favaloro E.J., Hill F.G., Holmberg L., Ingerslev J., Lee C.A., Lillicrap D., Mannucci P.M. (2006). Update on the pathophysiology and classification of von Willebrand disease: A report of the Subcommittee on von Willebrand Factor. J. Thromb. Haemost..

[30] Schneppenheim R., Hellermann N., Brehm M.A., Klemm U., Obser T., Huck V., Schneider S.W., Denis C.V., Tischer A., Auton M. (2019). The von Willebrand factor Tyr2561 allele is a gain-of-function variant and a risk factor for early myocardial infarction. Blood.

[31] Bongers T.N., de Maat M.P., van Goor M.L., Bhagwanbali V., van Vliet H.H., Gomez Garcia E.B., Dippel D.W., Leebeek F.W. (2006). High von Willebrand factor levels increase the risk of first ischemic stroke: Influence of ADAMTS13, inflammation, and genetic variability. Stroke.

[32] Crawley J.T., de Groot R., Xiang Y., Luken B.M., Lane D.A. (2011). Unraveling the scissile bond: How ADAMTS13 recognizes and cleaves von Willebrand factor. Blood.

[33] Lancellotti S., De Filippis V., Pozzi N., Peyvandi F., Palla R., Rocca B., Rutella S., Pitocco D., Mannucci P.M., De Cristofaro R. (2010). Formation of methionine sulfoxide by peroxynitrite at position 1606 of von Willebrand factor inhibits its cleavage by ADAMTS-13: A new prothrombotic mechanism in diseases associated with oxidative stress. Free Radic. Biol. Med..

[34] Chen J., Fu X., Wang Y., Ling M., McMullen B., Kulman J., Chung D.W., Lopez J.A. (2010). Oxidative modification of von Willebrand factor by neutrophil oxidants inhibits its cleavage by ADAMTS13. Blood.

[35] Fu X., Chen J., Gallagher R., Zheng Y., Chung D.W., Lopez J.A. (2011). Shear stress-induced unfolding of VWF accelerates oxidation of key methionine residues in the A1A2A3 region. Blood.

[36] De Filippis V., Lancellotti S., Maset F., Spolaore B., Pozzi N., Gambaro G., Oggianu L., Calo L.A., De Cristofaro R. (2012). Oxidation of Met1606 in von Willebrand factor is a risk factor for thrombotic and septic complications in chronic renal failure. Biochem. J..

[37] Jahroudi N., Lynch D.C. (1994). Endothelial-cell-specific regulation of von Willebrand factor gene expression. Mol. Cell. Biol..

[38] Liu J., Kanki Y., Okada Y., Jin E., Yano K., Shih S.C., Minami T., Aird W.C. (2009). A +220 GATA motif mediates basal but not endotoxin-repressible expression of the von Willebrand factor promoter in Hprt-targeted transgenic mice. J. Thromb. Haemost..

[39] Schwachtgen J.L., Janel N., Barek L., Duterque-Coquillaud M., Ghysdael J., Meyer D., Kerbiriou-Nabias D. (1997). Ets transcription factors bind and transactivate the core promoter of the von Willebrand factor gene. Oncogene.

[40] Wang X., Peng Y., Ma Y., Jahroudi N. (2004). Histone H1-like protein participates in endothelial cell-specific activation of the von Willebrand factor promoter. Blood.

[41] Jahroudi N., Ardekani A.M., Greenberger J.S. (1996). An NF1-like protein functions as a repressor of the von Willebrand factor promoter. J. Biol. Chem..

[42] Schwachtgen J.L., Remacle J.E., Janel N., Brys R., Huylebroeck D., Meyer D., Kerbiriou-Nabias D. (1998). Oct-1 is involved in the transcriptional repression of the von willebrand factor gene promoter. Blood.

[43] Peng Y., Jahroudi N. (2002). The NFY transcription factor functions as a repressor and activator of the von Willebrand factor promoter. Blood.

[44] Hough C., Cameron C.L., Notley C.R., Brown C., O’Brien L., Keightley A.M., Berber E., Lillicrap D. (2008). Influence of a GT repeat element on shear stress responsiveness of the VWF gene promoter. J. Thromb. Haemost..

[45] Shirodkar A.V., St Bernard R., Gavryushova A., Kop A., Knight B.J., Yan M.S., Man H.S., Sud M., Hebbel R.P., Oettgen P. (2013). A mechanistic role for DNA methylation in endothelial cell (EC)-enriched gene expression: Relationship with DNA replication timing. Blood.

[46] Ng C.J., Liu A., Ashworth K.J., Jones K.L., Di Paola J. (2018). Epigenetic Profiles of Primary Endothelial Cells from Patients with Low VWF Levels. Blood.

[47] Nakhaei-Nejad M., Farhan M., Mojiri A., Jabbari H., Murray A.G., Jahroudi N. (2019). Regulation of von Willebrand Factor Gene in Endothelial Cells That Are Programmed to Pluripotency and Differentiated Back to Endothelial Cells. Stem Cells.

[48] Peng Y., Jahroudi N. (2003). The NFY transcription factor inhibits von Willebrand factor promoter activation in non-endothelial cells through recruitment of histone deacetylases. J. Biol. Chem..

[49] Mojiri A., Stoletov K., Carrillo M.A., Willetts L., Jain S., Godbout R., Jurasz P., Sergi C.M., Eisenstat D.D., Lewis J.D. (2017). Functional assessment of von Willebrand factor expression by cancer cells of non-endothelial origin. Oncotarget.

[50] Mojiri A., Eisenstat D., Lewis J., Stoletov K., Simmen K., Jahroudi N. (2015). Epigenetic Modification of Von Willebrand Factor (VWF) Leads to its Expression in Cancer Cells with Increased Metastatic Activity. FASEB J..

[51] McEver R.P., Beckstead J.H., Moore K.L., Marshall-Carlson L., Bainton D.F. (1989). GMP-140, a platelet alpha-granule membrane protein, is also synthesized by vascular endothelial cells and is localized in Weibel-Palade bodies. J. Clin. Investig..

[52] Huang R.H., Wang Y., Roth R., Yu X., Purvis A.R., Heuser J.E., Egelman E.H., Sadler J.E. (2008). Assembly of Weibel-Palade body-like tubules from N-terminal domains of von Willebrand factor. Proc. Natl. Acad. Sci. USA.

[53] Haberichter S.L., Budde U., Obser T., Schneppenheim S., Wermes C., Schneppenheim R. (2010). The mutation N528S in the von Willebrand factor (VWF) propeptide causes defective multimerization and storage of VWF. Blood.

[54] Haberichter S.L., Allmann A.M., Jozwiak M.A., Montgomery R.R., Gill J.C. (2009). Genetic alteration of the D2 domain abolishes von Willebrand factor multimerization and trafficking into storage. J. Thromb. Haemost..

[55] Allen S., Abuzenadah A.M., Hinks J., Blagg J.L., Gursel T., Ingerslev J., Goodeve A.C., Peake I.R., Daly M.E. (2000). A novel von Willebrand disease-causing mutation (Arg273Trp) in the von Willebrand factor propeptide that results in defective multimerization and secretion. Blood.

[56] Hommais A., Stepanian A., Fressinaud E., Mazurier C., Meyer D., Girma J.P., Ribba A.S. (2006). Mutations C1157F and C1234W of von Willebrand factor cause intracellular retention with defective multimerization and secretion. J. Thromb. Haemost..

[57] Journet A.M., Saffaripour S., Wagner D.D. (1993). Requirement for both D domains of the propolypeptide in von Willebrand factor multimerization and storage. Thromb. Haemost..

[58] Voorberg J., Fontijn R., Calafat J., Janssen H., van Mourik J.A., Pannekoek H. (1993). Biogenesis of von Willebrand factor-containing organelles in heterologous transfected CV-1 cells. EMBO J..

[59] Wagner D.D., Saffaripour S., Bonfanti R., Sadler J.E., Cramer E.M., Chapman B., Mayadas T.N. (1991). Induction of specific storage organelles by von Willebrand factor propolypeptide. Cell.

[60] Metcalf D.J., Nightingale T.D., Zenner H.L., Lui-Roberts W.W., Cutler D.F. (2008). Formation and function of Weibel-Palade bodies. J. Cell Sci..

[61] Randi A.M., Smith K.E., Castaman G. (2018). von Willebrand factor regulation of blood vessel formation. Blood.

[62] Starke R.D., Ferraro F., Paschalaki K.E., Dryden N.H., McKinnon T.A., Sutton R.E., Payne E.M., Haskard D.O., Hughes A.D., Cutler D.F. (2011). Endothelial von Willebrand factor regulates angiogenesis. Blood.

[63] Hodivala-Dilke K. (2008). alphavbeta3 integrin and angiogenesis: A moody integrin in a changing environment. Curr. Opin. Cell Biol..

[64] Reynolds A.R., Hart I.R., Watson A.R., Welti J.C., Silva R.G., Robinson S.D., Da Violante G., Gourlaouen M., Salih M., Jones M.C. (2009). Stimulation of tumor growth and angiogenesis by low concentrations of RGD-mimetic integrin inhibitors. Nat. Med..

[65] Thomas M., Felcht M., Kruse K., Kretschmer S., Deppermann C., Biesdorf A., Rohr K., Benest A.V., Fiedler U., Augustin H.G. (2010). Angiopoietin-2 stimulation of endothelial cells induces alphavbeta3 integrin internalization and degradation. J. Biol. Chem..

[66] Soldi R., Mitola S., Strasly M., Defilippi P., Tarone G., Bussolino F. (1999). Role of alphavbeta3 integrin in the activation of vascular endothelial growth factor receptor-2. EMBO J..

[67] Maisonpierre P.C., Suri C., Jones P.F., Bartunkova S., Wiegand S.J., Radziejewski C., Compton D., McClain J., Aldrich T.H., Papadopoulos N. (1997). Angiopoietin-2, a natural antagonist for Tie2 that disrupts in vivo angiogenesis. Science.

[68] Markowska A.I., Liu F.T., Panjwani N. (2010). Galectin-3 is an important mediator of VEGF- and bFGF-mediated angiogenic response. J. Exp. Med..

[69] Saint-Lu N., Oortwijn B.D., Pegon J.N., Odouard S., Christophe O.D., de Groot P.G., Denis C.V., Lenting P.J. (2012). Identification of galectin-1 and galectin-3 as novel partners for von Willebrand factor. Arterioscler. Thromb. Vasc. Biol..

[70] Dzau V.J., Braun-Dullaeus R.C., Sedding D.G. (2002). Vascular proliferation and atherosclerosis: New perspectives and therapeutic strategies. Nat. Med..

[71] Kockx M.M., De Meyer G.R., Andries L.J., Bult H., Jacob W.A., Herman A.G. (1993). The endothelium during cuff-induced neointima formation in the rabbit carotid artery. Arterioscler. Thromb..

[72] De Meyer G.R., Hoylaerts M.F., Kockx M.M., Yamamoto H., Herman A.G., Bult H. (1999). Intimal deposition of functional von Willebrand factor in atherogenesis. Arterioscler. Thromb. Vasc. Biol..

[73] Bosmans J.M., Kockx M.M., Vrints C.J., Bult H., De Meyer G.R., Herman A.G. (1997). Fibrin(ogen) and von Willebrand factor deposition are associated with intimal thickening after balloon angioplasty of the rabbit carotid artery. Arterioscler. Thromb. Vasc. Biol..

[74] Giddings J.C., Banning A.P., Ralis H., Lewis M.J. (1997). Redistribution of von Willebrand factor in porcine carotid arteries after balloon angioplasty. Arterioscler. Thromb. Vasc. Biol..

[75] Qin F., Dardik H., Pangilinan A., Robinson J., Chuy J., Wengerter K. (2001). Remodeling and suppression of intimal hyperplasia of vascular grafts with a distal arteriovenous fistula in a rat model. J. Vasc. Surg..

[76] Qin F., Impeduglia T., Schaffer P., Dardik H. (2003). Overexpression of von Willebrand factor is an independent risk factor for pathogenesis of intimal hyperplasia: Preliminary studies. J. Vasc. Surg..

[77] Huang J., Roth R., Heuser J.E., Sadler J.E. (2009). Integrin alpha(v)beta(3) on human endothelial cells binds von Willebrand factor strings under fluid shear stress. Blood.

[78] Scheppke L., Murphy E.A., Zarpellon A., Hofmann J.J., Merkulova A., Shields D.J., Weis S.M., Byzova T.V., Ruggeri Z.M., Iruela-Arispe M.L. (2012). Notch promotes vascular maturation by inducing integrin-mediated smooth muscle cell adhesion to the endothelial basement membrane. Blood.

[79] Lagrange J., Worou M.E., Michel J.B., Raoul A., Didelot M., Muczynski V., Legendre P., Plenat F., Gauchotte G., Lourenco-Rodrigues M.D. (2021). The VWF/LRP4/alphaVbeta3-axis represents a novel pathway regulating proliferation of human vascular smooth muscle cells. Cardiovasc. Res..

[80] Tomer A. (2004). Human marrow megakaryocyte differentiation: Multiparameter correlative analysis identifies von Willebrand factor as a sensitive and distinctive marker for early (2N and 4N) megakaryocytes. Blood.

[81] Deutsch V.R., Tomer A. (2013). Advances in megakaryocytopoiesis and thrombopoiesis: From bench to bedside. Br. J. Haematol..

[82] Patel S.R., Hartwig J.H., Italiano J.E. (2005). The biogenesis of platelets from megakaryocyte proplatelets. J. Clin. Investig..

[83] Balduini A., Pallotta I., Malara A., Lova P., Pecci A., Viarengo G., Balduini C.L., Torti M. (2008). Adhesive receptors, extracellular proteins and myosin IIA orchestrate proplatelet formation by human megakaryocytes. J. Thromb. Haemost..

[84] Dunois-Larde C., Capron C., Fichelson S., Bauer T., Cramer-Borde E., Baruch D. (2009). Exposure of human megakaryocytes to high shear rates accelerates platelet production. Blood.

[85] Chang M., Nakagawa P.A., Williams S.A., Schwartz M.R., Imfeld K.L., Buzby J.S., Nugent D.J. (2003). Immune thrombocytopenic purpura (ITP) plasma and purified ITP monoclonal autoantibodies inhibit megakaryocytopoiesis in vitro. Blood.

[86] McMillan R., Wang L., Tomer A., Nichol J., Pistillo J. (2004). Suppression of in vitro megakaryocyte production by antiplatelet autoantibodies from adult patients with chronic ITP. Blood.

[87] Nurden P., Debili N., Vainchenker W., Bobe R., Bredoux R., Corvazier E., Combrie R., Fressinaud E., Meyer D., Nurden A.T. (2006). Impaired megakaryocytopoiesis in type 2B von Willebrand disease with severe thrombocytopenia. Blood.

[88] Estevez B., Du X. (2017). New Concepts and Mechanisms of Platelet Activation Signaling. Physiology.

[89] Varga-Szabo D., Braun A., Nieswandt B. (2009). Calcium signaling in platelets. J. Thromb. Haemost..

[90] Hathaway D.R., Adelstein R.S. (1979). Human platelet myosin light chain kinase requires the calcium-binding protein calmodulin for activity. Proc. Natl. Acad. Sci. USA.

[91] Li Z., Zhang G., Marjanovic J.A., Ruan C., Du X. (2004). A platelet secretion pathway mediated by cGMP-dependent protein kinase. J. Biol. Chem..

[92] Shattil S.J., Brass L.F. (1987). Induction of the fibrinogen receptor on human platelets by intracellular mediators. J. Biol. Chem..

[93] Kehrel B., Wierwille S., Clemetson K.J., Anders O., Steiner M., Knight C.G., Farndale R.W., Okuma M., Barnes M.J. (1998). Glycoprotein VI is a major collagen receptor for platelet activation: It recognizes the platelet-activating quaternary structure of collagen, whereas CD36, glycoprotein IIb/IIIa, and von Willebrand factor do not. Blood.

[94] Rosado J.A., Meijer E.M., Hamulyak K., Novakova I., Heemskerk J.W., Sage S.O. (2001). Fibrinogen binding to the integrin alpha(IIb)beta(3) modulates store-mediated calcium entry in human platelets. Blood.

[95] Andonegui G., Kerfoot S.M., McNagny K., Ebbert K.V., Patel K.D., Kubes P. (2005). Platelets express functional Toll-like receptor-4. Blood.

[96] Ishihara H., Connolly A.J., Zeng D., Kahn M.L., Zheng Y.W., Timmons C., Tram T., Coughlin S.R. (1997). Protease-activated receptor 3 is a second thrombin receptor in humans. Nature.

[97] Zarpellon A., Celikel R., Roberts J.R., McClintock R.A., Mendolicchio G.L., Moore K.L., Jing H., Varughese K.I., Ruggeri Z.M. (2011). Binding of alpha-thrombin to surface-anchored platelet glycoprotein Ib(alpha) sulfotyrosines through a two-site mechanism involving exosite I. Proc. Natl. Acad. Sci. USA.

[98] Du X. (2007). Signaling and regulation of the platelet glycoprotein Ib-IX-V complex. Curr. Opin. Hematol..

[99] Ju L., Chen Y., Zhou F., Lu H., Cruz M.A., Zhu C. (2015). Von Willebrand factor-A1 domain binds platelet glycoprotein Ibalpha in multiple states with distinctive force-dependent dissociation kinetics. Thromb. Res..

[100] Zhang W., Deng W., Zhou L., Xu Y., Yang W., Liang X., Wang Y., Kulman J.D., Zhang X.F., Li R. (2015). Identification of a juxtamembrane mechanosensitive domain in the platelet mechanosensor glycoprotein Ib-IX complex. Blood.

[101] Ju L., Lou J., Chen Y., Li Z., Zhu C. (2015). Force-Induced Unfolding of Leucine-Rich Repeats of Glycoprotein Ibalpha Strengthens Ligand Interaction. Biophys. J..

[102] Dai K., Bodnar R., Berndt M.C., Du X. (2005). A critical role for 14-3-3zeta protein in regulating the VWF binding function of platelet glycoprotein Ib-IX and its therapeutic implications. Blood.

[103] Ju L., Chen Y., Xue L., Du X., Zhu C. (2016). Cooperative unfolding of distinctive mechanoreceptor domains transduces force into signals. eLife.

[104] Wu Y., Asazuma N., Satoh K., Yatomi Y., Takafuta T., Berndt M.C., Ozaki Y. (2003). Interaction between von Willebrand factor and glycoprotein Ib activates Src kinase in human platelets: Role of phosphoinositide 3-kinase. Blood.

[105] Yin H., Liu J., Li Z., Berndt M.C., Lowell C.A., Du X. (2008). Src family tyrosine kinase Lyn mediates VWF/GPIb-IX-induced platelet activation via the cGMP signaling pathway. Blood.

[106] Yin H., Stojanovic A., Hay N., Du X. (2008). The role of Akt in the signaling pathway of the glycoprotein Ib-IX induced platelet activation. Blood.

[107] Riba R., Oberprieler N.G., Roberts W., Naseem K.M. (2006). Von Willebrand factor activates endothelial nitric oxide synthase in blood platelets by a glycoprotein Ib-dependent mechanism. J. Thromb. Haemost..

[108] Li Z., Xi X., Du X. (2001). A mitogen-activated protein kinase-dependent signaling pathway in the activation of platelet integrin alpha IIbbeta3. J. Biol. Chem..

[109] Li Z., Zhang G., Feil R., Han J., Du X. (2006). Sequential activation of p38 and ERK pathways by cGMP-dependent protein kinase leading to activation of the platelet integrin alphaIIb beta3. Blood.

[110] Smolenski A. (2012). Novel roles of cAMP/cGMP-dependent signaling in platelets. J. Thromb. Haemost..

[111] Watanabe N., Bodin L., Pandey M., Krause M., Coughlin S., Boussiotis V.A., Ginsberg M.H., Shattil S.J. (2008). Mechanisms and consequences of agonist-induced talin recruitment to platelet integrin alphaIIbbeta3. J. Cell Biol..

[112] Rhee S.G., Bae Y.S. (1997). Regulation of phosphoinositide-specific phospholipase C isozymes. J. Biol. Chem..

[113] Prakriya M., Lewis R.S. (2015). Store-Operated Calcium Channels. Physiol. Rev..

[114] Berliner S., Niiya K., Roberts J.R., Houghten R.A., Ruggeri Z.M. (1988). Generation and characterization of peptide-specific antibodies that inhibit von Willebrand factor binding to glycoprotein IIb-IIIa without interacting with other adhesive molecules. Selectivity is conferred by Pro1743 and other amino acid residues adjacent to the sequence Arg1744-Gly1745-Asp1746. J. Biol. Chem..

[115] Li S., Wang Z., Liao Y., Zhang W., Shi Q., Yan R., Ruan C., Dai K. (2010). The glycoprotein Ibalpha-von Willebrand factor interaction induces platelet apoptosis. J. Thromb. Haemost..

[116] van Schooten C.J., Shahbazi S., Groot E., Oortwijn B.D., van den Berg H.M., Denis C.V., Lenting P.J. (2008). Macrophages contribute to the cellular uptake of von Willebrand factor and factor VIII in vivo. Blood.

[117] Castro-Nunez L., Dienava-Verdoold I., Herczenik E., Mertens K., Meijer A.B. (2012). Shear stress is required for the endocytic uptake of the factor VIII-von Willebrand factor complex by macrophages. J. Thromb. Haemost..

[118] Rastegarlari G., Pegon J.N., Casari C., Odouard S., Navarrete A.M., Saint-Lu N., van Vlijmen B.J., Legendre P., Christophe O.D., Denis C.V. (2012). Macrophage LRP1 contributes to the clearance of von Willebrand factor. Blood.

[119] Smith (2010). Novel Associations of Multiple Genetic Loci with Plasma Levels of Factor VII, Factor VIII, and von Willebrand Factor: The CHARGE (Cohorts for Heart and Aging Research in Genome Epidemiology) (vol 121, pg 1382, 2010). Circulation.

[120] Wohner N., Muczynski V., Mohamadi A., Legendre P., Proulle V., Ayme G., Christophe O.D., Lenting P.J., Denis C.V., Casari C. (2018). Macrophage scavenger receptor SR-AI contributes to the clearance of von Willebrand factor. Haematologica.

[121] Pegon J.N., Kurdi M., Casari C., Odouard S., Denis C.V., Christophe O.D., Lenting P.J. (2012). Factor VIII and von Willebrand factor are ligands for the carbohydrate-receptor Siglec-5. Haematologica.

[122] Grewal P.K., Uchiyama S., Ditto D., Varki N., Le D.T., Nizet V., Marth J.D. (2008). The Ashwell receptor mitigates the lethal coagulopathy of sepsis. Nat. Med..

[123] Ward S.E., O’Sullivan J.M., Drakeford C., Aguila S., Jondle C.N., Sharma J., Fallon P.G., Brophy T.M., Preston R.J.S., Smyth P. (2018). A novel role for the macrophage galactose-type lectin receptor in mediating von Willebrand factor clearance. Blood.

[124] Rydz N., Swystun L.L., Notley C., Paterson A.D., Riches J.J., Sponagle K., Boonyawat B., Montgomery R.R., James P.D., Lillicrap D. (2013). The C-type lectin receptor CLEC4M binds, internalizes, and clears von Willebrand factor and contributes to the variation in plasma von Willebrand factor levels. Blood.

[125] Swystun L.L., Lai J.D., Notley C., Georgescu I., Paine A.S., Mewburn J., Nesbitt K., Schledzewski K., Geraud C., Kzhyshkowska J. (2018). The endothelial cell receptor stabilin-2 regulates VWF-FVIII complex half-life and immunogenicity. J. Clin. Investig..

[126] Pendu R., Terraube V., Christophe O.D., Gahmberg C.G., de Groot P.G., Lenting P.J., Denis C.V. (2006). P-selectin glycoprotein ligand 1 and beta2-integrins cooperate in the adhesion of leukocytes to von Willebrand factor. Blood.

[127] Petri B., Broermann A., Li H., Khandoga A.G., Zarbock A., Krombach F., Goerge T., Schneider S.W., Jones C., Nieswandt B. (2010). von Willebrand factor promotes leukocyte extravasation. Blood.

[128] Ayme G., Adam F., Legendre P., Bazaa A., Proulle V., Denis C.V., Christophe O.D., Lenting P.J. (2017). A Novel Single-Domain Antibody Against von Willebrand Factor A1 Domain Resolves Leukocyte Recruitment and Vascular Leakage During Inflammation-Brief Report. Arterioscler. Thromb. Vasc. Biol..

[129] Hillgruber C., Steingraber A.K., Poppelmann B., Denis C.V., Ware J., Vestweber D., Nieswandt B., Schneider S.W., Goerge T. (2014). Blocking von Willebrand factor for treatment of cutaneous inflammation. J. Invest. Dermatol..

[130] Mizgerd J.P., Kubo H., Kutkoski G.J., Bhagwan S.D., Scharffetter-Kochanek K., Beaudet A.L., Doerschuk C.M. (1997). Neutrophil emigration in the skin, lungs, and peritoneum: Different requirements for CD11/CD18 revealed by CD18-deficient mice. J. Exp. Med..

[131] Braun L.J., Stegmeyer R.I., Schafer K., Volkery S., Currie S.M., Kempe B., Nottebaum A.F., Vestweber D. (2020). Platelets docking to VWF prevent leaks during leukocyte extravasation by stimulating Tie-2. Blood.

[132] Braun L.J., Zinnhardt M., Vockel M., Drexler H.C., Peters K., Vestweber D. (2019). VE-PTP inhibition stabilizes endothelial junctions by activating FGD5. EMBO Rep..

[133] Kawecki C., Lenting P.J., Denis C.V. (2017). von Willebrand factor and inflammation. J. Thromb. Haemost..

[134] McEver R.P., Cummings R.D. (1997). Role of PSGL-1 binding to selectins in leukocyte recruitment. J. Clin. Investig..

[135] Michaux G., Pullen T.J., Haberichter S.L., Cutler D.F. (2006). P-selectin binds to the D’-D3 domains of von Willebrand factor in Weibel-Palade bodies. Blood.

[136] Denis C.V., Andre P., Saffaripour S., Wagner D.D. (2001). Defect in regulated secretion of P-selectin affects leukocyte recruitment in von Willebrand factor-deficient mice. Proc. Natl. Acad. Sci. USA.

[137] Noone S., Schubert R., Fichtlscherer S., Hilberg T., Alesci S., Miesbach W. (2021). Endothelial Function in Patients with Von Willebrand Disease. Clin. Appl. Thromb. Hemost..

[138] Bernardo A., Ball C., Nolasco L., Choi H., Moake J.L., Dong J.F. (2005). Platelets adhered to endothelial cell-bound ultra-large von Willebrand factor strings support leukocyte tethering and rolling under high shear stress. J. Thromb. Haemost..

[139] Zhang J., Tecson K.M., McCullough P.A. (2020). Endothelial dysfunction contributes to COVID-19-associated vascular inflammation and coagulopathy. Rev. Cardiovasc. Med..

[140] Mannucci P.M. (1998). von Willebrand factor: A marker of endothelial damage?. Arterioscler. Thromb. Vasc. Biol..

[141] Pober J.S., Sessa W.C. (2007). Evolving functions of endothelial cells in inflammation. Nat. Rev. Immunol..

[142] Zirka G., Robert P., Tilburg J., Tishkova V., Maracle C.X., Legendre P., van Vlijmen B.J.M., Alessi M.C., Lenting P.J., Morange P.E. (2021). Impaired adhesion of neutrophils expressing Slc44a2/HNA-3b to VWF protects against NETosis under venous shear rates. Blood.

[143] Grassle S., Huck V., Pappelbaum K.I., Gorzelanny C., Aponte-Santamaria C., Baldauf C., Grater F., Schneppenheim R., Obser T., Schneider S.W. (2014). von Willebrand factor directly interacts with DNA from neutrophil extracellular traps. Arterioscler. Thromb. Vasc. Biol..

[144] Sandoval-Perez A., Berger R.M.L., Garaizar A., Farr S.E., Brehm M.A., Konig G., Schneider S.W., Collepardo-Guevara R., Huck V., Radler J.O. (2020). DNA binds to a specific site of the adhesive blood-protein von Willebrand factor guided by electrostatic interactions. Nucleic Acids Res..

[145] Ward C.M., Tetaz T.J., Andrews R.K., Berndt M.C. (1997). Binding of the von Willebrand factor A1 domain to histone. Thromb. Res..

[146] Savchenko A.S., Borissoff J.I., Martinod K., De Meyer S.F., Gallant M., Erpenbeck L., Brill A., Wang Y., Wagner D.D. (2014). VWF-mediated leukocyte recruitment with chromatin decondensation by PAD4 increases myocardial ischemia/reperfusion injury in mice. Blood.

[147] Dasgupta S., Repesse Y., Bayry J., Navarrete A.M., Wootla B., Delignat S., Irinopoulou T., Kamate C., Saint-Remy J.M., Jacquemin M. (2007). VWF protects FVIII from endocytosis by dendritic cells and subsequent presentation to immune effectors. Blood.

[148] Takahashi N., Udagawa N., Suda T. (1999). A new member of tumor necrosis factor ligand family, ODF/OPGL/TRANCE/RANKL, regulates osteoclast differentiation and function. Biochem. Biophys. Res. Commun..

[149] Shahbazi S., Lenting P.J., Fribourg C., Terraube V., Denis C.V., Christophe O.D. (2007). Characterization of the interaction between von Willebrand factor and osteoprotegerin. J. Thromb. Haemost..

[150] Zannettino A.C., Holding C.A., Diamond P., Atkins G.J., Kostakis P., Farrugia A., Gamble J., To L.B., Findlay D.M., Haynes D.R. (2005). Osteoprotegerin (OPG) is localized to the Weibel-Palade bodies of human vascular endothelial cells and is physically associated with von Willebrand factor. J. Cell. Physiol..

[151] Chollet M.E., Brouland J.P., Bal dit Sollier C., Bauduer F., Drouet L., Bellucci S. (2010). Evidence of a colocalisation of osteoprotegerin (OPG) with von Willebrand factor (VWF) in platelets and megakaryocytes alpha granules. Studies from normal and grey platelets. Br. J. Haematol..

[152] Simonet W.S., Lacey D.L., Dunstan C.R., Kelley M., Chang M.S., Luthy R., Nguyen H.Q., Wooden S., Bennett L., Boone T. (1997). Osteoprotegerin: A novel secreted protein involved in the regulation of bone density. Cell.

[153] Tsuda E., Goto M., Mochizuki S., Yano K., Kobayashi F., Morinaga T., Higashio K. (1997). Isolation of a novel cytokine from human fibroblasts that specifically inhibits osteoclastogenesis. Biochem. Biophys. Res. Commun..

[154] Burgess T.L., Qian Y., Kaufman S., Ring B.D., Van G., Capparelli C., Kelley M., Hsu H., Boyle W.J., Dunstan C.R. (1999). The ligand for osteoprotegerin (OPGL) directly activates mature osteoclasts. J. Cell Biol..

[155] Hsu H., Lacey D.L., Dunstan C.R., Solovyev I., Colombero A., Timms E., Tan H.L., Elliott G., Kelley M.J., Sarosi I. (1999). Tumor necrosis factor receptor family member RANK mediates osteoclast differentiation and activation induced by osteoprotegerin ligand. Proc. Natl. Acad. Sci. USA.

[156] Baud’huin M., Duplomb L., Teletchea S., Charrier C., Maillasson M., Fouassier M., Heymann D. (2009). Factor VIII-von Willebrand factor complex inhibits osteoclastogenesis and controls cell survival. J. Biol. Chem..

[157] Emery J.G., McDonnell P., Burke M.B., Deen K.C., Lyn S., Silverman C., Dul E., Appelbaum E.R., Eichman C., DiPrinzio R. (1998). Osteoprotegerin is a receptor for the cytotoxic ligand TRAIL. J. Biol. Chem..

[158] Wiley S.R., Schooley K., Smolak P.J., Din W.S., Huang C.P., Nicholl J.K., Sutherland G.R., Smith T.D., Rauch C., Smith C.A. (1995). Identification and characterization of a new member of the TNF family that induces apoptosis. Immunity.

[159] Mochizuki S., Soejima K., Shimoda M., Abe H., Sasaki A., Okano H.J., Okano H., Okada Y. (2012). Effect of ADAM28 on carcinoma cell metastasis by cleavage of von Willebrand factor. J. Natl. Cancer Inst..

[160] Terraube V., Pendu R., Baruch D., Gebbink M.F., Meyer D., Lenting P.J., Denis C.V. (2006). Increased metastatic potential of tumor cells in von Willebrand factor-deficient mice. J. Thromb. Haemost..

[161] Feinauer M.J., Schneider S.W., Berghoff A.S., Robador J.R., Tehranian C., Karreman M.A., Venkataramani V., Solecki G., Grosch J.K., Gunkel K. (2021). Local blood coagulation drives cancer cell arrest and brain metastasis in a mouse model. Blood.

[162] Goertz L., Schneider S.W., Desch A., Mayer F.T., Koett J., Nowak K., Karampinis I., Bohlmann M.K., Umansky V., Bauer A.T. (2016). Heparins that block VEGF-A-mediated von Willebrand factor fiber generation are potent inhibitors of hematogenous but not lymphatic metastasis. Oncotarget.

[163] Patmore S., Dhami S.P.S., O’Sullivan J.M. (2020). Von Willebrand factor and cancer; metastasis and coagulopathies. J. Thromb. Haemost..

[164] Karagiannis G.S., Saraon P., Jarvi K.A., Diamandis E.P. (2014). Proteomic signatures of angiogenesis in androgen-independent prostate cancer. Prostate.

[165] Bauer A.T., Suckau J., Frank K., Desch A., Goertz L., Wagner A.H., Hecker M., Goerge T., Umansky L., Beckhove P. (2015). von Willebrand factor fibers promote cancer-associated platelet aggregation in malignant melanoma of mice and humans. Blood.

[166] Kerk N., Strozyk E.A., Poppelmann B., Schneider S.W. (2010). The mechanism of melanoma-associated thrombin activity and von Willebrand factor release from endothelial cells. J. Invest. Dermatol..

[167] Xu Y., Pan S., Liu J., Dong F., Cheng Z., Zhang J., Qi R., Zang Q., Zhang C., Wang X. (2017). GATA3-induced vWF upregulation in the lung adenocarcinoma vasculature. Oncotarget.

[168] John A., Robador J.R., Vidal Y.S.S., Houdek P., Wladykowski E., Gunes C., Bolenz C., Schneider S.W., Bauer A.T., Gorzelanny C. (2020). Urothelial Carcinoma of the Bladder Induces Endothelial Cell Activation and Hypercoagulation. Mol. Cancer Res..

[169] Pilch J., Habermann R., Felding-Habermann B. (2002). Unique ability of integrin alpha(v)beta 3 to support tumor cell arrest under dynamic flow conditions. J. Biol. Chem..

[170] Suter C.M., Hogg P.J., Price J.T., Chong B.H., Ward R.L. (2001). Identification and characterisation of a platelet GPIb/V/IX-like complex on human breast cancers: Implications for the metastatic process. Jpn. J. Cancer Res..

[171] Eppert K., Wunder J.S., Aneliunas V., Kandel R., Andrulis I.L. (2005). von Willebrand factor expression in osteosarcoma metastasis. Mod. Pathol..

[172] Liu G., Ren Y.M. (2010). [Effect of von Willebrand factor on the biological characteristics of colorectal cancer cells]. Zhonghua Wei Chang Wai Ke Za Zhi.

[173] Liu Y., Wang X., Li S., Hu H., Zhang D., Hu P., Yang Y., Ren H. (2014). The role of von Willebrand factor as a biomarker of tumor development in hepatitis B virus-associated human hepatocellular carcinoma: A quantitative proteomic based study. J. Proteom..

[174] Yang A.J., Wang M., Wang Y., Cai W., Li Q., Zhao T.T., Zhang L.H., Houck K., Chen X., Jin Y.L. (2018). Cancer cell-derived von Willebrand factor enhanced metastasis of gastric adenocarcinoma. Oncogenesis.

